# MoS_2_-functionalized sulfonated polystyrene for adsorption of rhodamine B

**DOI:** 10.1039/d5ra07598j

**Published:** 2026-01-05

**Authors:** Kashmala Khaliq, Adil Khan, Shabnam Shahida, Ramzan Akhtar, Mohsin Ali Raza Anjum, Iqra Rafiq, Muhammad Rehan, Rashid Nazir Qureshi, Sajid Iqbal, Muhammad Saifullah

**Affiliations:** a Department of Chemistry, University of Poonch Rawalakot AJK Pakistan shabnamshahida01@gmail.com; b Chemistry Division, Pakistan Institute of Nuclear Science and Technology (PINSTECH) Nilore 45650 Islamabad Pakistan saifi.551@gmail.com; c Department of Chemistry, Government College University Faisalabad (GCUF) Faisalabad Pakistan; d Photovoltaic Research Department, Korea Institute of Energy Research Daejeon South Korea; e Central Analytical Facility Division, Pakistan Institute of Nuclear Science and Technology (PINSTECH) Nilore 45650 Islamabad Pakistan; f Department of Nuclear and Quantum Engineering, KAIST 291 Deahak-ro, Yuseong-gu Daejeon 34141 Republic of Korea sajid1@kaist.ac.kr

## Abstract

A novel MoS_2_-incorporated sulfonated polystyrene composite (MOSP) is developed for the selective removal of rhodamine B (RhB) from aqueous solutions. The materials are characterized using SEM-EDS, XRD, Raman spectroscopy, FTIR, CHNS elemental analysis, BET, and TGA, while UV-visible spectrophotometry and ICP-OES are employed to quantify RhB and interfering metal ions. Batch adsorption experiments are carried out to optimize pH, contact time, dye concentration, adsorbent dosage, and temperature. Kinetic studies indicate that chemisorption, described by the pseudo-second-order model, is the dominant mechanism, with rapid adsorption reaching equilibrium within one hour. Under optimal conditions, MOSP achieves a maximum adsorption capacity of 400 mg g^−1^ for RhB, as described by the Langmuir isotherm. Thermodynamic analysis confirmed that the process is spontaneous and exothermic. MOSP retains >90% RhB removal efficiency even in the presence of alkali, alkaline-earth, and transition metal ions. Owing to its high selectivity, facile synthesis, rapid kinetics, large capacity, cost-effectiveness, and stability, MOSP shows strong potential for commercial-scale RhB removal from wastewater.

## Abstract 2

A novel MoS_2_-incorporated sulfonated polystyrene composite (MOSP) is developed for the selective removal of rhodamine B (RhB) from aqueous solutions.

## Graphical abstract

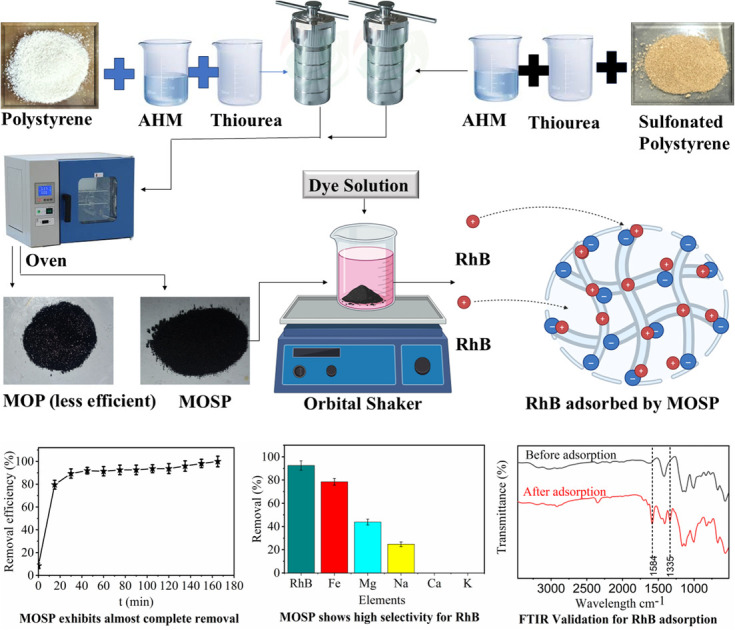

## 1. Introduction

Organic dyes are extensively used in a wide range of industries, including textiles, pigments, cosmetics, paper manufacturing, food processing, and pharmaceuticals.^1^ However, their widespread application, coupled with improper disposal practices, poses significant environmental and health hazards. Wastewater discharged from dye manufacturing and application processes is a major contributor to water pollution, threatening both ecosystems and human health. Numerous dyes and their degradation byproducts are known to have toxic, mutagenic, carcinogenic, or teratogenic properties, further intensifying these concerns.^2^

Among synthetic dyes, rhodamine B (RhB) is a water-soluble, basic red dye belonging to the xanthene class. Due to its coloration and fluorescence, RhB is extensively used as a coloring agent in textiles and dietary products, and works as a broadly recognized fluorescent tracer in water analysis.^3^ RhB lowers the photosynthesis of aquatic plants.^4^ Moreover, it poses a severe health risk if consumed by humans or animals and may lead to irritation of the eyes, skin, and respiratory system.^5^ Experimental research has shown the reproductive, carcinogenic, and developmental toxicity, as well as chronic toxicity and neurotoxicity of RhB in both humans and animals. However, with appropriate RhB mitigation strategies, these detrimental impacts can be reduced or entirely precluded.^6^

Given the escalating pollution of water resources, it is necessary to employ efficient purification techniques to remove toxic pollutants.^7–9^ Conventional wastewater remediation techniques, including coagulation, filtration, sedimentation, aeration, and chemical flocculation, exhibit a notable capacity to remove textile dye pollutants. However, these techniques suffer from several limitations, including the generation of hazardous byproducts, high energy consumption, offensive odors, and the need for large treatment areas. These drawbacks underscore the need for alternative technologies to improve wastewater remediation. Among emerging alternatives, adsorption has drawn considerable attention due to its superior efficiency. It is widely regarded as a cost-effective, simple, and versatile approach capable of removing a broad spectrum of pollutants.^10^

Over the past few decades, researchers have developed and modified a wide range of novel adsorbents for dye removal from wastewater. These include clays and zeolites, activated carbon, biosorbents, industrial byproducts, agricultural wastes, metal–organic frameworks (MOFs), layered double hydroxide-based materials, various polymers, and other innovative materials.^11–18^ Additionally, two-dimensional (2D) nanomaterials have garnered considerable attention, attributed to their distinctive properties and extensive potential applications. This category includes a broad spectrum of materials, comprising 2D honeycomb-structured silicon, hexagonal boron nitride, graphene, and transition metal dichalcogenides, namely molybdenum disulfide and tungsten disulfide.^19^ Among them, molybdenum disulfide (MoS_2_), a two-dimensional layered inorganic compound, has emerged as a multifunctional material with applications in environmental sensing, photocatalysis, membrane technology, and adsorption.^20,21^ In terms of adsorptive performance, MoS_2_ exhibits strong interactions with dye molecules.^22^ This contributes to MoS_2_'s high adsorption capacity, driven by a large number of active sites and rapid adsorption kinetics due to their easy accessibility.^23^ Furthermore, MoS_2_ is chemically stable in aqueous environments under mild acidic conditions, though it can dissolve in hot concentrated sulfuric acid and aqua regia.^24^

The adsorption of RhB dye using MoS_2_ remains relatively underexplored, highlighting the need for further studies. Yang *et al.*^21^ synthesized MoS_2_/MIL-101 (MoS_2_ nanosheet-coated MIL-101 chromium terephthalate hybrid) having a high adsorption capacity, *i.e.* 344.8 mg g^−1^, and rapid adsorption kinetics. In another study, Li *et al.*^25^ fabricated MoS_2_*via* a straightforward hydrothermal method with various sulfur sources, and the results show that MoS_2_–CH_4_N_2_S exhibited the highest adsorption capacity of 136.99 mg g^−1^ for RhB. In a subsequent study, Ma *et al.*^26^ developed MoS_2_-PDA@FRC (MoS_2_ nanosheet modified biochar) composite that shows an adsorption capacity of 58.11 mg g^−1^. Furthermore, Fang *et al.*^27^ synthesized MoS_2_ nanopowder through the hydrothermal method, and the analysis of adsorption performance demonstrates that MoS_2_ adsorption capacity for RhB is 210.24 mg g^−1^.

Pure MoS_2_ nanopowder tends to agglomerate and is also difficult to recover after the adsorption has been completed. Recent advancements have examined the loading of MoS_2_ nanosheets with different porous supports, including carbon dodecahedrons and TiO_2_ nanostructures, utilizing their surface area to facilitate MoS_2_ growth.^28–30^ However, their low surface porosity suppresses MoS_2_ loading. Therefore, designing support with high surface porosity is pivotal to improving MoS_2_ growth and overall adsorption performance.^21^ To prevent the MoS_2_ nanopowder agglomeration and improve adsorption efficiency, polystyrene divinylbenzene is used as a support material. Its highly porous structure promotes the uniform dispersion of MoS_2_ particles, increasing the accessibility of active sites and maximizing the removal efficiency of RhB from the aqueous solution.

Although various polymer and MoS_2_-based adsorbents have been used for water treatment applications, to the best of our knowledge, integration of MoS_2_ into sulfonated polystyrene has not been reported yet. This study explains the development of novel adsorbent material by incorporating MoS_2_ into pristine and sulfonated polystyrene divinylbenzene (PS-DVB) resins *via* an easy, cost-effective, and simple hydrothermal method. This *in situ* procedure enables the homogenous growth of MoS_2_ within the polystyrene host matrix. The resulting MoS_2_-incorporated pristine and sulfonated PS-DVB is assessed for adsorptive removal of RhB from the aqueous solution. The MoS_2_-impregnated sulfonated PS-DVB demonstrates a significantly higher removal efficiency and adsorption capacity for RhB. The presence of the sulfonic acid group assures the maximum MoS_2_ loading on sulfonated PS-DVB, which increases its surface area and maximizes the active sites, thereby enhancing its adsorption capacity for RhB.

## 2. Materials and methods

### 2.1. Materials

The experimental study is conducted using following materials, hexaammonium heptamolybdate (AHM) ((NH_4_)_6_Mo_7_O_24_·4H_2_O) (BDH, 98%), thiourea (CN_2_H_2_S) (Riedal-dehaen, 98.5%), rhodamine B (C_28_H_31_CIN_2_O_3_) (RhB) (Riedal-dehaen, 99%), styrene (98%), divinyl benzene (DVB) (Sigma Aldrich, 85%), dichloromethane (DCM) (99%), gelatin pyruvate (Merck, 99%), cyclohexanone (Merck, 99%), benzoyl peroxide (BPO) (Sigma Aldrich, 98%), gum Arabic (98%), sulfuric acid (H_2_SO_4_) (Merck, 97.5%), deionized water (DI), hydrochloric acid (HCl, 99%), and sodium hydroxide (NaOH, 98%).

### 2.2 Synthesis of polystyrene- and sulfonated polystyrene resin-supported MoS_2_

To synthesize polystyrene-divinylbenzene (PS-DVB), the previously reported suspension polymerization method with slight modifications is used.^31^ 3 g of gelatin and 3 g of gum Arabic are dissolved in 250 mL of DI water to prepare the aqueous phase. This phase is combined with the organic phases comprising styrene, BPO, DVB, and cyclohexanone. The mixture is transferred to a reactor and stirred at 350 rpm at 95 °C for 6 h. After polymerization, polystyrene resin (P) is filtered, washed, and dried at 60 °C. For functionalization, 10 g of P is swollen in DCM in a beaker overnight, and treated with H_2_SO_4_ at 85 °C for 5 h, following which sulfonated polystyrene (SP) is washed and dried at 60 °C. 2.5 g of P and SP are added to two Teflon-lined stainless steel reaction vessels. In a separate beaker, 0.704 g of AHM is dissolved in 50 mL of DI water. Additionally, 2.1 g of thiourea is dissolved in 15 mL of a separate beaker.^32^ Both solutions are combined, and the pH of the solution is measured, which is around 4. The two separate mixtures are transferred to two Teflon-lined stainless steel autoclave reaction vessels with P and PS and heated at 180 °C for 6 h. After the reaction is completed, centrifugation is performed, and the pH of the solution is measured, which is around 8. The resulting samples are dried in an oven at 60 °C overnight. MoS_2_-incorporated pristine polystyrene (MOP) and MoS_2_-incorporated sulfonated polystyrene (MOSP) are further characterized, and their synthesis schematic representation is presented in Fig. S1.

### 2.3 Characterization

Field emission scanning electron microscopy (FE-SEM) (SU8230, Hitachi, Japan) is used to analyze the surface morphology of the sample. To determine the structural characteristics, particularly phase composition and degree of crystallinity, X-ray diffractometer (EQUINOX 3000 X-ray) (XRD) with Cu K_α_ (*λ* = 1.5408 Å) radiation is used in the scan range of 10–80° and step size of 0.05°. A Raman spectrometer (Horiba XploRA™ Plus) with a 532 nm wavelength is used to investigate the vibrational modes of the MoS_2_. For the detection of functional groups, Fourier-transform infrared spectra (Thermo Scientific, NICOLET IS50) (FTIR) is used in a frequency range of 4000–400 cm^−1^. An elemental analyzer (ThermoFlash 2000, Italia) is used to determine the C, H, N, and S concentrations in the samples. The specific surface area is determined through N_2_ adsorption/desorption (3Flex, Micromeritics) after degassing the samples at 100 °C for 12 h using Brunauer–Emmett–Teller (BET). The thermogravimetric analysis (TGA) (METLER TOLEDO GA/SDTA851) is done to evaluate the thermal stability of the material. To examine surface charge characteristics, a zeta potential analyzer is used. The quantification of RhB in the adsorption study is performed using a UV-visible spectrophotometer (Hitachi U-2900) by taking into account the absorption peak at approximately 552 nm. An inductively coupled plasma-optical emission spectrometer (ICP-OES) (Thermo Scientific iCAP 6000) (ICP-OES) is used to analyze the concentration of interfering metal ions used in the selectivity experiment. To capture the optical images, an optical microscope (Olympus Japan) is used.

### 2.4 Adsorption experiment

10 mg L^−1^ of RhB dye solution is prepared for the batch adsorption experiment, to which 0.01 g of the adsorbent is added. The mixture is stirred at 250 rpm for 2 h using an orbital shaker to ensure sorption equilibrium. After adsorption, the supernatant is subjected to centrifugation and filtration, and the residual RhB concentration in the supernatant is examined using a UV-visible spectrophotometer.

Eqn (1) and (2) are used to determine the removal efficiency (%) and adsorption capacity (mg g^−1^) of the prepared adsorbent.1
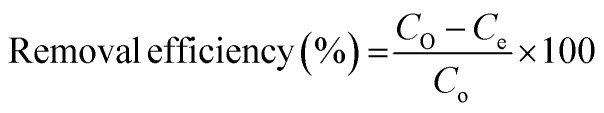
2
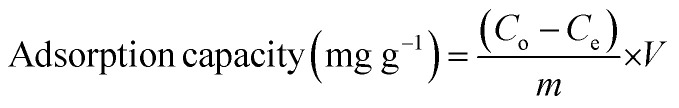
where *C*_o_ and *C*_e_ represent the initial and equilibrium concentration (mg L^−1^) of RhB dye, while *m* denotes the mass of adsorbent (g), and *V* is the volume of solution (L).

To evaluate the adsorption behavior of RhB dye on the best adsorbent, *i.e.* MOSP, several experiments are conducted to study key contributing factors, including pH, contact time, adsorbent dose, dye concentration, and temperature effect.

#### 2.4.1 The pH-dependent adsorption

The influence of pH on adsorption is determined by mixing 0.01 g of the adsorbent in 100 mL of 10 mg L^−1^ RhB solution at 25 °C for 75 min. The pH of the solution is adjusted between 1 and 11 using 0.1 M HCl and 0.1 M NaOH solutions. After the reaction, the adsorbent is separated by centrifugation and subsequently filtered using a filter paper. The remaining dye concentration in the solution is quantified using a UV-visible spectrophotometer.

#### 2.4.2 Solid-to-liquid ratio

To determine the dosage effect on dye removal, different amounts of adsorbent (0.01, 0.05, 0.1, 0.15, and 0.2 g L^−1^) are added to a 10 mg L^−1^ RhB solution. The mixtures are agitated at 250 rpm for 75 min, and the residual dye concentration is determined.

#### 2.4.3 Adsorption kinetics

To analyze the kinetic performance of MOSP, the pseudo-first-order (PFO), pseudo-second-order (PSO), intraparticle diffusion model (IPD), and Elovich kinetic models are employed. In the PFO kinetic model, the adsorption rate is directly proportional to the number of unoccupied (available) adsorption sites, while in the PSO model, the rate of adsorption depends on the square of available active sites. The mathematical expressions of the PFO and PSO kinetic models are presented in eqn (3) and (4).

3 ln (*q*_e_ − *q*_*t*_) = ln (*q*_e_) − *k*_1_*t*

4
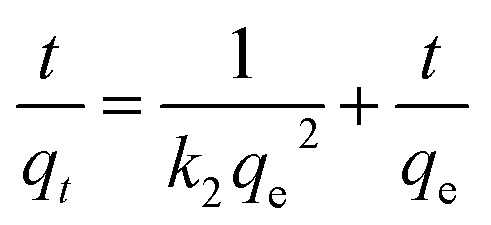


The kinetic parameters, *k*_1_ (min^−1^) is the PFO rate constant, *k*_2_ (mg g^−1^min^−1^) is the PSO rate constant, *q*_*t*_ and *q*_e_ signify adsorption capacity (mg g^−1^) at equilibrium.^33–36^

Additionally, in the IPD model, a three-step mechanism is used to describe the adsorption process. The 1st is bulk diffusion, where the molecules of adsorbate migrate toward the external surface of the adsorbent from the liquid phase, the second is film diffusion, in which at the solid–liquid interface, the movement of these molecules involves across the boundary layer, and the third is pore diffusion, involving the diffusion of molecules into the internal pores of the adsorbent. Moreover, the Elovich kinetic model is extensively used to explain the chemisorption processes of heterogeneous systems. It is based on two assumptions concerning adsorbate and adsorbent interaction. (1) There is a possibility that the adsorbent surface is heterogeneous. (2) As the adsorption progresses, the activation energy needed for adsorption is likely to increase.^37,38^ The IPD and Elovich model can be described by eqn (5) and (6),

5 *q*_*t*_ = *k*_id_*t*^0.5^ + *C*

6 *q*_*t*_ = 1/*β* ln(*αβ*) + 1/*β* ln *t*

where, *k*_id_ (mg g^−1^ min^−0.5^) is the IPD rate constant, *α* (mg g^−1^ min^−1^) is the Elovich rate constant, *C* is the intercept, and *β* is the desorption constant at any given time (*t*).

To evaluate the adsorption kinetics, 0.01 g of MOSP is mixed with 10 mg L^−1^ rhodamine solution and stirred on an orbital shaker for 2 h and 45 min. Samples are collected after 0, 15, 30, 45, 60, 75, 90, 105, 120, 135, 150, and 165 minutes, subsequently, subjected to centrifugation, filtration, and analysis to determine the residual RhB concentration.

#### 2.4.4 Adsorption isotherms

The different isotherm models, Langmuir, Freundlich, and Temkin, are used to describe the adsorption mechanism and to evaluate the maximum adsorption capacity of MOSP. The Langmuir model explains monolayer adsorption on the adsorbent surface, generally presuming that the surface comprises a finite number of uniform active sites.^31^ This model can be expressed as:7
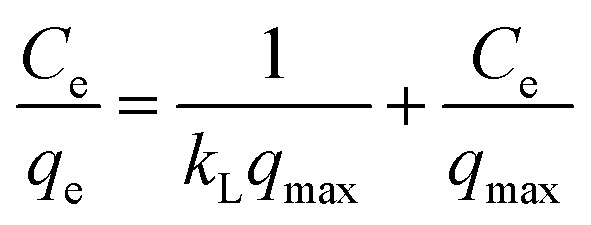
8
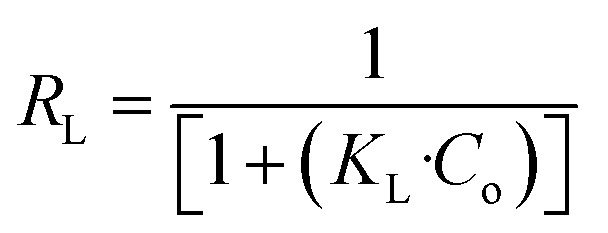


For the Langmuir isotherm equation (eqn (7)), Ce(mg L^−1^) represents the equilibrium concentration of adsorbate, *q*_e_ (mg g^−1^) is the adsorption capacity, *q*_max_ is the maximum adsorption capacity, and *K*_L_ denotes the Langmuir adsorption constant. Langmuir parameter, *R*_L_ (eqn (8)) is the separation factor, used to assess the adsorption process favourability. The value of *R*_L_ below 1 indicates a favorable adsorption process, and the value of *R*_L_ above 1 depicts that the adsorption process is unfavorable.

The Freundlich model explains the adsorption on a heterogeneous surface and represents multilayer adsorption.^39^ This model is represented mathematically in eqn (9).9
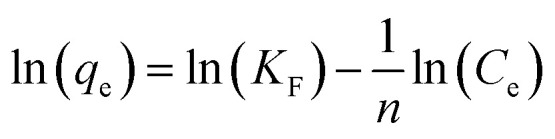
In the Freundlich isotherm. eqn (9), *K*_F_ is the Freundlich isotherm constant, *q*_e_ (mg g^−1^) denotes the adsorption capacity, and *n* represents the favorability of adsorption.

Furthermore, the Temkin isotherm model describes that the heat of adsorption decreases linearly with the increase in surface coverage due to the interaction between adsorbate and adsorbent, and can be expressed as eqn (10),

10 *q*_e_ = *B*_T_ln A_T_ + *B*_T_ln*C*_e_

where *A*_T_ (g^−1^) is the Temkin binding constant, and *B*_T_ denotes the heat of adsorption.^40^

In this study, RhB solutions with 5, 10, 30, 50, 70, and 90 mg L^−1^ concentrations are prepared to examine the influence of initial dye concentration on adsorption. 10 mg of adsorbent is added to each dye solution, and the mixtures are agitated for 1 h. The residual rhodamine concentration is analyzed to determine the adsorption capacity at different initial concentrations.

#### 2.4.5 Thermodynamic studies

The thermodynamic laws as presented in eqn (11) and (12) are utilized to examine the thermodynamic parameters of the adsorption process, such as standard entropy change (Δ*S*°), standard enthalpy change (Δ*H*°), and standard Gibbs free energy (Δ*G*°). In the van't Hoff equation (eqn (11)), Δ*S*° and Δ*H*° are calculated from the slope and intercept by plotting a graph between ln *K*_c_ against *T*. *R* denotes the general gas constant in eqn (11), having a value of 8.314 J K^−1^ mol^−1^, and *T* represents the temperature in kelvin (*K*).11
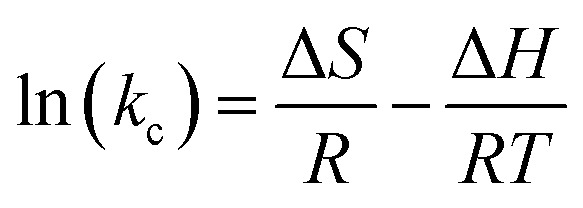

12 Δ*G* = Δ*H* − *T*Δ*S*

The effect of temperature on rhodamine adsorption is evaluated at 298, 303, 318, 328, and 338 K.

#### 2.4.6 Selectivity experiment

To examine the effect of metal ions on the adsorption capacity of MOSP for RhB removal, an equimolar mixture of dye, monovalent (Na^+^, K^+^), and divalent (Ca^2+^, Fe^2+^, Mg^2+^) ions is prepared in a single batch. The experimental conditions, pH, adsorbent dose, contact time, and shaking speed are maintained constant.

## 3. Results and discussions

### 3.1 Morphological analysis

**Fig. 1:**
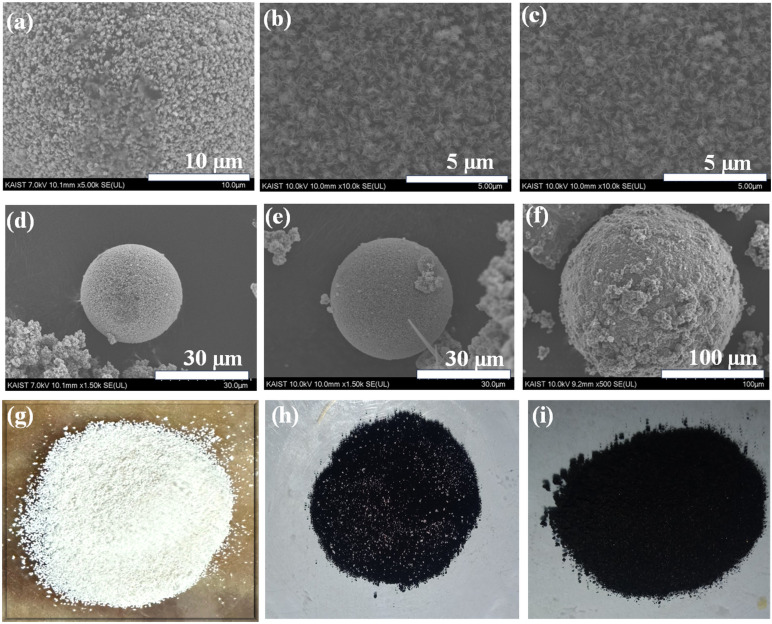
SEM surface images at higher magnification of pristine PS (a), MOP (b), and MOSP (c), and SEM images at lower magnification of PS (d), MOP (e), and MOSP (f), and digital photographs of PS (g), MOP (h), and MOSP (i).

The SEM images present a clear comparison of pristine PS, MOP, and MOSP, signifying the differences in surface features and morphology. In Fig. 1d, PS beads exhibit a smooth and spherical morphology, a typical morphological feature obtained in suspension polymerization, where surface tension during droplet nucleation results in well-defined beads.^41^ At slightly higher magnification (Fig. 1a), a relatively smooth and fine texture is seen on the surface of pristine PS. This smoothness signifies the absence of structural modification and any inorganic feature on the polymer surface. In MOP, when MoS_2_ is incorporated into polystyrene (Fig. 1e), the spherical geometry of the bead is retained, indicating that the MoS_2_ addition does not disrupt the polymer's morphology. However, significant changes are observed as the surface becomes roughened after the incorporation of MoS_2_ on the polymer surface.^42^ The surface image (Fig. 1b) of MOP at higher magnification exhibits nanosheet-like features across the bead that are consistent with MoS_2_ morphology.^32,43^ The growth of MoS_2_ on the polymer bead seems to be uniform in Fig. 1b, but the photograph of MOP (Fig. 1h) shows that MoS_2_ growth on MOP beads on a larger scale is not homogeneous, and some of the beads remained uncoated. This reveals that while the introduction of MoS_2_ did occur, the absence of functional groups on the polymer led to localized aggregation and limited anchoring of MoS_2_, which can be seen in the digital photographs (Fig. 1h), optical images (Fig. S4d), and elemental mapping (Fig. S3) of MOP. A pronounced change is observed when MoS_2_ is integrated into sulfonated polystyrene (Fig. 1f). The spherical shape remains intact, but the surface roughness increases, with signs of irregularities that indicate enhanced deposition and plausible surface degradation due to sulfonation.^44^ Sulfonation substantially changes surface texture and facilitates favorable conditions for MoS_2_ loading. The sulfonic acid functional groups in sulfonated polystyrene act as strong anchoring sites for Mo during synthesis, promoting uniform growth of MoS_2_.^31^ Although the surface morphology of MOSP at higher magnification (Fig. 1c) is similar to that of MOP (Fig. 1b), the coverage of MoS_2_ on sulfonated polystyrene is significantly greater, as further supported by Fig. 1i and S4e, EDX spectrum (Fig. S2a), and elemental mapping (Fig. S2b) which clearly show the uniform coating of MoS_2_ on the sulfonated polystyrene in comparison to the pristine polystyrene beads.

### 3.2 Structural and vibrational characterization

**Fig. 2:**
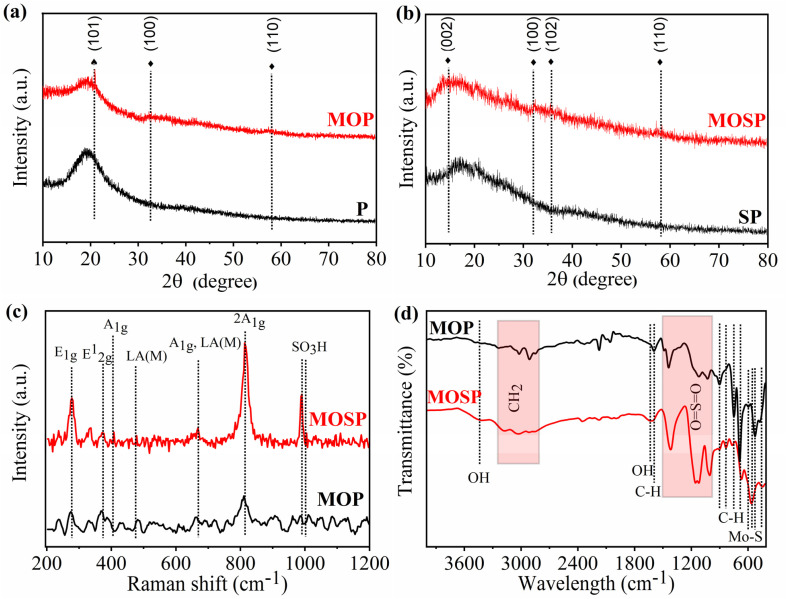
XRD patterns of P, MOP (a), and SP, MOSP (b) taken in *θ*–2*θ* mode, Raman spectra of MOP and MOSP (c), and FTIR spectra of MOP and MOSP (d).

XRD analysis is performed for structural analysis of the synthesized materials P, MOP, SP, and MOSP. Fig. 2a shows a change in the XRD patterns of P and MOP. The sample P shows a broad hump at a 2*θ* value of 19.08°, which is indicative of the amorphous nature of polystyrene, as it was also reported in our previous study.^31^ Upon growing MoS_2_ on polystyrene, a distinct peak emerges at 2*θ* = 20.82°, corresponding to the (101) plane of Mo_2_S_3_ in a monoclinic crystal system (PDF card 01-081-2031, space group *P*2_1_/*m*). Additional minor peaks seen at 2*θ* = 32.50° and 58.16° correspond to the (100) and (110) planes of hexagonal MoS_2_ (PDF card 00-037-1492, space group *P*6_3_/*mmc*), respectively. The low intensities indicate that MoS_2_ is present in a nanocrystalline state,^45^ while the Mo_2_S_3_ peak suggests a mixed phase rather than pure MoS_2_.

Fig. 2b shows that SP exhibits a broader hump at 2*θ* = 16.87°, indicating the absence of long-range crystalline order. This is expected for sulfonated polystyrene, as sulfonation disrupts the ordered packing of polystyrene chains by introducing polar acidic groups, thereby reinforcing the amorphous character.^31^ However, the XRD spectrum of the MOSP (*i.e.* sample with MoS_2_ grown on SP) demonstrates a composite structure with nanocrystalline and amorphous features. A characteristic peak of MoS_2_ appears at a 2*θ* value of 14.38°, arising from the (002) plane of MoS_2_, suggesting successful incorporation of layered MoS_2_ into the sulfonated polystyrene. The (002) plane of MoS_2_ in MOSP presents a larger interplanar spacing (*d* = 0.615 nm), which enhances the adsorption process by increasing accessible sites and promoting interlayer diffusion.^46^ The broad hump correlated with the polymer matrix is visibly sustained and overlaps with the crystalline peaks of MoS_2_. Additional small peaks are observed at around 32.73°, 35.98°, and 57.92°, corresponding to the planes (100), (102), and (110), respectively, of a hexagonal crystal system of MoS_2_, which matches with the PDF card number 00-037-1492, having space group *P*6_3_/*mmc*.

These peaks are consistent with the peaks present in the XRD spectrum of pristine MoS_2_ (Fig. S5) at 2*θ* values of 13.89°, 32.50°, 35.75°, and 57.48°, arises from the planes (002), (100), (102), and (110), respectively, indicating MoS_2_ with the hexagonal crystal system (PDF card number 00-002-1133, space group *P*63/*mmc*). The pristine MoS_2_ (Fig. S5) also exhibits a small diffraction peak of Mo_2_S_3_, which appears at a 2*θ* value of 20.42°, corresponding to the (101) plane in the monoclinic crystal system (PDF card number 01-081-2031, space group *P*2_1_/*m*^1^), and is present in MOP. This indicates the mixed phase of pristine MoS_2_. Further, MOSP peaks are relatively broad and low in intensity, revealing poorly crystalline MoS_2_. In MOSP, sulfonation functionalities facilitate the formation of pure-phase MoS_2_, unlike MOP and sole MoS_2_, presumably due to the polar SO_3_H groups in SP, facilitating uniform nucleation sites and promoting interaction with molybdenum precursors. Sulfonation also improves surface hydrophilicity, which can enhance MoS_2_ growth on the sulfonated matrix. Furthermore, the main amorphous hump observed in SP extends towards lower 2*θ*, due to a broad peak emerging from the (002) plane of MoS_2_. Overall, the SP amorphous framework supports the growth of phase-pure nanocrystalline MoS_2_ on it. This also points out that the polymer chemical nature is decisive in controlling the phase purity of grown MoS_2_. In essence, a transition from a comparatively phase impure MoS_2_ in MOP to a phase-pure MoS_2_ in MOSP, primarily influenced by the stronger interaction of the MoS_2_ with the sulfonated matrix.

The resonant Raman scattering of MoS_2_ in both the composite materials has been analyzed, taking into account both the zone centers first-order Raman process (FOR) and the second-order Raman process (SOR), which are augmented through the interaction of phonon modes with electronic transitions correlated with excitonic states.^47^Fig. 2c presents the comparative Raman findings for studying the electronic and vibrational properties of MOSP and MOP. Among the vibrational modes of MoS_2_, A_1g_, E_1g_, and two E_2g_ are Raman active.^48^ Spectra of both MOP and MOSP show characteristic peaks of MoS_2_, E^1^_2g_ (379 cm^−1^), which coincide with the in-plane vibration of sulfur and molybdenum atoms. MOSP exhibits a peak at 406 cm^−1^, which corresponds to A_1g_ out-of-plane vibration of the sulfur atoms, prominently the first-order Raman active mode, thereby corroborating the successful impregnation of MoS_2_ in MOSP and MOP.^32,49,50^ This mode is considerably stronger in MOSP, and a clear separation between A_1g_ and E^1^_2g_ validates layered MoS_2_ formation.^51–53^ The presence of these peaks (E^1^_2g_, A_1g_) and the absence of J bands (J_1_, J_2_, J_3_) of the 1T phase validates the 2H (hexagonal) phase of MoS_2_.^54–57^ Another band at around 278 cm^−1^ in both spectra, associated with the E_1g_ vibration, is commonly forbidden in back scattering from the basal plane.^58^ The MOSP shows additional Raman peaks resulting from strong electron–phonon interaction. The band near 460–470 cm^−1^ arises from a second-order process comprising longitudinal acoustic phonons (LA(M)). These peaks are typically assigned to multiphonon bands that involve LA(M) and other phonons positioned at the M point.^59^ The spectral band at 665 cm^−1^ emerges from the cumulative contribution of the A_1g_ and LA(M) phonon modes. Another second-order overtone mode, located around 816 cm^−1^, is attributed to the A_1g_ mode (2 A_1g_ region).^60^ These Raman peaks are supported by the peaks present in pristine MoS_2_ (Fig. S6). In addition to the MoS_2_ modes, MOSP exhibits peaks around 990–1003 cm^−1^. These peaks are ascribed to the vibrations in the polymer backbone, such as symmetric stretching of the SO_3_H group and aromatic ring vibrations.^61^ The increased intensity and slight shift of these peaks in MOSP highlight the interaction between the SO_3_H group and MoS_2_. This interaction improves charge transfer between the organic and inorganic phases, which can be advantageous for adsorption applications.

The FTIR spectra (Fig. 2d) of MOP and MOSP exhibit significant differences, highlighting successful functionalization and discrete interaction between the functional groups in the polymer matrix and MoS_2_. A broad band at around 3439 cm^−1^, along with the bending vibrations at 1627 cm^−1^, is observed in MOSP and associated with O–H stretching and water bending vibration, respectively.^60,62^ These are characteristic hydroxyl group peaks, typically introduced during the sulfonation process. Their absence in MOP supports the fact that these functional groups emanate from sulfonation. In the range between 2800 cm^−1^ and 3100 cm^−1^, several bands appear in both spectra, indicating the symmetric and asymmetric vibrations of CH_2_ groups.^63^ This region is evident as the structural integrity of the polystyrene backbone remains intact after hydrothermal synthesis of both MOP and MOSP. These peaks are in close agreement with the peaks reported by Brijmohan *et al.*^64^ Besides this, at 1584 cm^−1^, a minor peak appears in MOP due to in-plane C–H stretching vibrations of an aromatic ring.^32,63^ The sharp peaks at approximately 1406 cm^−1^, 1165 cm^−1^, 1128 cm^−1^, and 1024 cm^−1^ in both the MOP and MOSP spectra correspond to the symmetric and asymmetric vibrations of the O=S=O groups.^32,34,65^ The higher intensity and slightly shifted positions of these peaks to lower wavelengths in MOSP are due to the strong hydrogen bonding interaction of MoS_2_ with sulfonic acid groups. These results are consistent with sulfonated polystyrene resin as described by Pandey *et al.*,^66^ who demonstrated similar features of SO_3_H groups appended onto benzene rings, and a study by Hazarika *et al.*,^67^ which explained the shifting of peaks in the composite. Additionally, at 840 cm^−1^, a small peak is observed in MOSP, with the blue shift seen for MOP at this position. Yang *et al.*^65^ reported the peak at the same position ascribed to the out-of-plane C–H vibrations for the para substitution of the benzene ring. Furthermore, the peaks appear at around 756 cm^−1^ and 675 cm^−1^ are present in both samples and are associated with the out-of-plane C–H bending vibrations.^64^ The peak at 756 cm^−1^ corresponds to monosubstituted benzene rings, whereas the peak at 675 cm^−1^ is typically assigned to styrene ring deformations. These peaks are slightly red-shifted in MOSP, which likely arises from hydrogen-bonding interactions between MoS_2_ and SO_3_H.^67,68^ In the fingerprint region, peaks are observed at around 594, 530, and 462 cm^−1^ in both spectra, which are assignable to the Mo–S stretching vibrations, substantiating the presence of MoS_2_ in the polystyrene matrix.^32,61,69^ These findings are similar to those reported by Singh *et al.*,^61^ who also observed Mo–S vibrations within this range in layered MoS_2_. However, a slight blue shift is observed in MOSP peaks in this region, as these shifts indicate strong Mo–SO_3_H interactions, which increase Mo–S bond strength and raise the vibrational frequency. It is worth highlighting that a slight shift in MOSP peaks is due to the interaction between the MoS_2_ and SO_3_H groups.

### 3.3 Elemental and surface area analysis

The CHNS data (Table 1) of all the samples, including P, MOP, SP, and MOSP, exhibit important compositional insights pertinent to the sulfonated and MoS_2_ impregnated polymer matrix. In the P, 7.08 moles of carbon, 6.94 moles of hydrogen, and sulfur content below the detection limit are observed, which is typical for a polystyrene divinylbenzene matrix. In the MOP sample, carbon and hydrogen contents slightly decreased, along with the emergence of sulfur. The slight but noticeable reduction in hydrogen and carbon content in MOP compared to P is ascribed to the introduction of MoS_2_ in the polystyrene, which adds sulfur and dilutes the organic content.^31^ Furthermore, hydrothermal treatment typically causes slight chemical changes to the polymer, resulting in a slight reduction of carbon and hydrogen content. The appearance of 0.149 moles of sulfur confirms the successful incorporation of MoS_2_. Contrarily, the pronounced drop of carbon content in SP to 2.97 moles is expected during sulfonation because of the modification or partial substitution of aromatic rings through the introduction of SO_3_H groups.^31^ Furthermore, the higher sulfur content of 0.33 moles indicates the successful sulfonation of polystyrene beads.

**Table 1:** Summary of C, H, and S concentrations in P, MOP, SP, and MOSP determined through CHNS analysis

Sample ID	C (moles)	H (moles)	S (moles)
P	7.08	6.94	ND
MOP	6.10	6.00	0.149
SP	2.97	5.75	0.33
MOSP	2.42	4.38	0.610

In the MOSP, the carbon content further drops to 2.42 moles and the hydrogen content to 4.38 moles, implying the introduction of MoS_2_ and the structural rearrangement or possible degradation of the polystyrene host during the hydrothermal process. The highest value of sulfur content of 0.610 moles suggests the combined presence of SO_3_H groups and molybdenum sulfides. The significant increase in sulfur content from SP to MOSP validates the notion that sulfonated polystyrene provides a conducive environment for MoS_2_ loading, likely through binding with the SO_3_H sites.

The surface area, pore volume, and pore size values of MOP and MOSP determined through BET analysis are reported in Table S1 and Fig. S7. MOSP has a surface area of 2.4068 m^2^ g^−1^, which is slightly higher compared with the 1.6350 m^2^ g^−1^ of MOP, confirming the successful growth of MoS_2_ on sulfonated resin. The pore volume and pore size in MOSP are reduced in comparison to MOP (Table S1), indicating that the introduction of MoS_2_ partially fills the macropores of sulfonated polystyrene and creates new mesopores.^70^

### 3.4 Thermal stability analysis

**Fig. 3:**
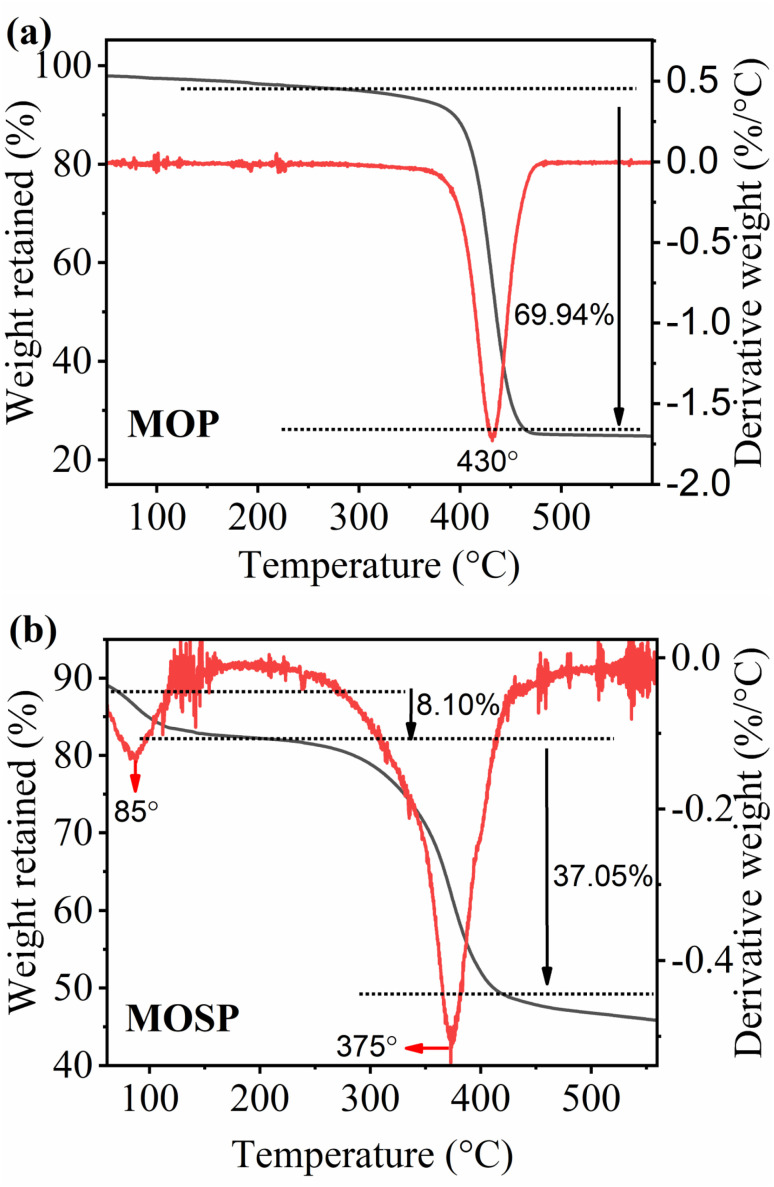
TGA-DTG profiles of MOP (a) and MOSP (b).

The TGA curves of MOSP and MOP (Fig. 3) show noticeable thermal decomposition behaviors resulting from the different chemical structure of sulfonated and pristine polystyrene. A minor weight loss of 8.10% occurs for MOSP (Fig. 3b) at 85 °C, which is associated with the release of retained moisture or physically adsorbed water in the hydrophilic sulfonated matrix, and the initial decomposition of SO_3_H groups.^71^ Due to the strong chemical interaction of MoS_2_ with sulfonated polystyrene, the degradation proceeds gradually, and a char-like network forms instead of undergoing rapid depolymerization.^72^ After 350 °C, moderate weight loss (37.05%) is observed at around 375 °C, and a substantial leftover mass remains until 600 °C. This high residual mass reflects the high thermal stability of MoS_2_. In contrast, MOP (Fig. 3a) shows a considerably different behavior. The polymer chain remains stable up to 400 °C with almost no mass loss, consistent with the non-ionic and hydrophobic nature of polystyrene. However, once decomposition begins, it occurs as a one-step rapid decomposition which is a typical characteristic of polystyrene. At around 430 °C, a major mass loss of 69.94% is observed, which aligns with the volatilization of species produced as a result of polymer breakdown and abrupt chain scission.^73^ After degradation, the residue is considerably lower compared with MOSP, consistent with the findings that sulfonated polystyrene has high and uniform dispersion of MoS_2_. The sulfonation shifts the degradation pathway into a multi-step process, resulting in greatly enhanced thermal stability. This confirms that the combination of sulfonated polystyrene and MoS_2_ enhanced thermal robustness, consistent with the findings of metal-oxides-reinforced sulfonated resins.^31^

### 3.5 Adsorption study

**Fig. 4:**
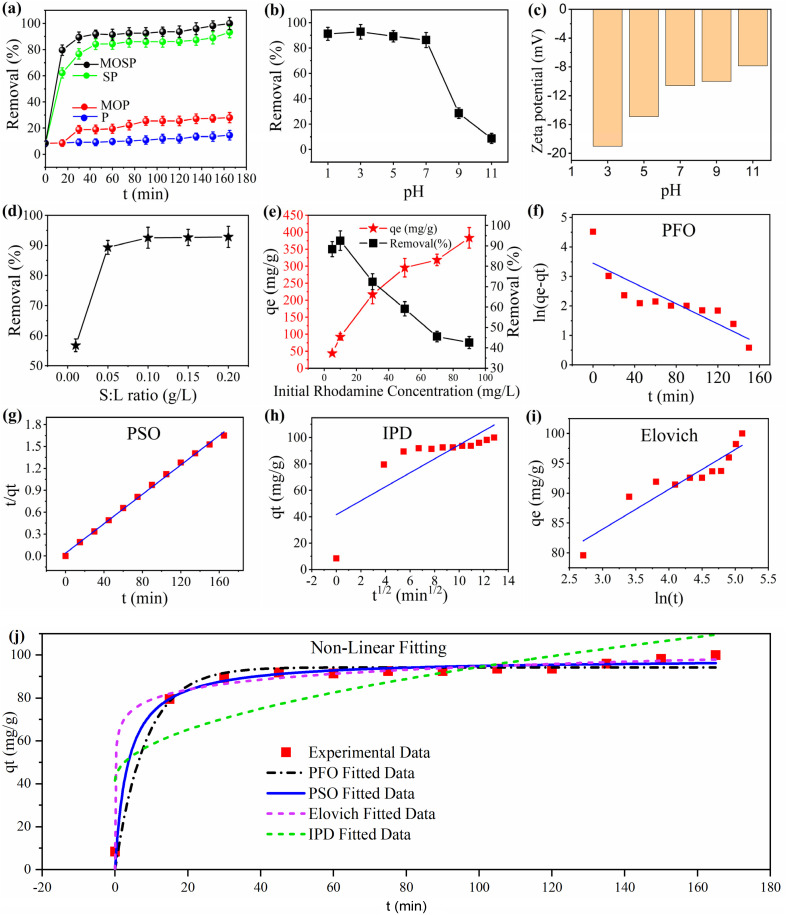
(a) Comparative adsorptive removal of RhB by PS, MOP, SP, and MOSP *versus* time, (b) effect of pH ranging from 1 to 11 on adsorption of RhB onto MOSP (Co = 10 mg L^−1^, contact time = 60 min), (c) zeta potential (mV) of MOSP at different pH values, (d) effect of adsorbent dose in the range of 0.01 to 0.2 g L^−1^ (Co = 10 mg L^−1^, contact time = 60 min), (e) effect of RhB concentration (Co = 10 mg L^−1^, contact time = 60 min, shaking speed = 250 rpm, and S : L ratio = 0.1 g L^−1^), and adsorption kinetics (f) PFO model fitting, (g) PSO model fitting, (h) IPD model fitting, (i) Elovich model fitting, and (j) non-linear curve-fitted PFO, PSO, IPD, Elovich model fitting (Co = 10 mg L^−1^, contact time = 60 min, shaking speed = 250 rpm, S : L ratio = 0.1 g L^−1^).

The comparative rhodamine (RhB) dye removal kinetics by different adsorbent materials (P, MOP, SP, and MOSP) are presented in Fig. 4a. P is the pristine base polymer, with minimal (≥10%) dye removal efficiency. MOP marginally enhanced the removal efficiency, exceeding around 25.50% after 2 h. However, this increased removal efficiency is insufficient for dye removal, implying that in the absence of surface functionalization, polystyrene does not permit sufficient MoS_2_ loading. SP showed a considerable improvement in removal efficiency, achieving over 62.20% removal in the first 15 min and 80.80% dye removal within 45 min of contact time. This demonstrates that sulfonation increases adsorption by incorporating a functional group that enhances adsorbent interaction with the dye molecule. However, the most pronounced performance is observed with MOSP, as the results demonstrate an initial fast adsorption phase, at which removal efficiency increases rapidly from 8.41 to 79.58% in the first 15 min, and increases to greater than 90.50% within 45 min. This shows a strong interaction between RhB and the adsorbent, with numerous active sites facilitating rapid uptake. Equilibrium is achieved within 1 h. With increasing time, the removal rate slows down, reaching 100% by 165 min. This indicates that primary active sites are occupied, and the system has attained saturation. This demonstrates the highest removal efficiency of MOSP, with rapid initial uptake proceeding by a slower equilibrium phase, establishing it as a promising candidate for effective wastewater treatment applications.

The best removal performance is observed with MOSP, which attains approximately 79.60% removal within the first 15 min and >90% in 45 min. The high removal efficiency and rapid kinetics of MOSP can be ascribed to the combined effect of sulfonation and MoS_2_ incorporation. Sulfonation increases surface properties and enhances the material's affinity for RhB dye molecules, and MoS_2_ increases surface area, which results in higher adsorption efficiency as more active sites are available for adsorption. The amorphous nature of MOSP is due to the sulfonated polystyrene host, which provides accessible binding sites for RhB interaction.^31^ Additionally, the nano-crystallinity of MoS_2_ nanosheets increases surface area, allowing more interaction points with RhB.^19^ The higher adsorption efficiency is directly correlated with the structural characteristics of MOSP, as evidenced by XRD data (Fig. 2b), confirming that the disordered nature improves the adsorption capability of MOSP composite. Therefore, MOSP exhibited the best performance among all the tested materials and is subsequently selected for further study.

#### 3.5.1 Effect of pH

The solution pH serves a fundamental role in the adsorption process, as it substantially influences the adsorbent's surface charge as well as chemical behavior and speciation of the adsorbate. A change in pH can alter the ionization state of functional groups on the material surface, thereby affecting the adsorption mechanism and efficiency by influencing the electrostatic interaction between the target species and the adsorbent.^70^Fig. 4b depicts the effect of pH on RhB removal using the MOSP. The removal efficiency alters significantly at different pH values, with optimal removal (>90%) at pH 3, and the minimum removal (<10%) observed at pH 11. At very low pH < 3, excessive proton activity may cause partial oxidation, resulting in sulfur removal. This trend highlights the critical role of pH in the adsorption mechanism. RhB dye contains a nitrogen atom with a lone pair, which is protonated under acidic conditions, promoting its interaction with the adsorbent. Moreover, the adsorbent's functional group, specifically the SO_3_H and MoS_2_ sites, promotes strong electrostatic interactions with a cationic dye molecule. Additionally, the incorporated MoS_2_ layers aid in adsorption through surface active sites and π–π interaction, which are more efficient under acidic conditions.^21^ Conversely, in basic conditions, RhB markedly transforms into zwitterion species. Above pH 3, the removal efficiency begins to decrease due to the portion of the RhB molecule that converts into the zwitterionic form, and these species are electrostatically less interactive, reducing the overall adsorption potential at higher pH values. Additionally, RhB in zwitterionic form has a higher propensity to aggregate and form dimers, and may face stearic hindrance.^21,74^ In the case of MOSP, a pore blocking mechanism could arise if aggregated species hinder the resin pores or impair accessibility to active sites on the MoS_2_ surface, resulting in minimal dye removal at higher pH values, particularly at pH 9 and 11. So, the soft acid–base interaction principle and ion exchange mechanism primarily govern the adsorption process. Furthermore, the zeta potential findings (Fig. 4c and S8(a–e)) indicate that the adsorbent retains negative charge among all tested pH values, and the point of zero charge is not detected. This behavior results from the inherently MoS_2_ surface and ionization of sulfonic acid groups on the polystyrene, both of which, even under the acidic conditions, ensure surface negativity. Different studies on MoS_2_ and sulfonated polystyrene also report negative zeta potential across all pH ranges.^75,76^ Therefore, strong electrostatic attraction towards the cationic dye is due to the MOSP surface negative charge at low pH. Overall, the results demonstrate that acidic conditions, particularly pH 3, are more favorable for the adsorption of RhB, and it ensure the structural stability of MOSP.

#### 3.5.2 Effect of adsorbent dose

Investigating the effect of adsorbent dosage is essential in adsorption studies to assess both performance and cost-effectiveness. Fig. 4d shows the variation in RhB removal efficiency with MOSP dosage. At a low dose of 0.01 g L^−1^, the removal rate is 56.79%, indicating that the limited adsorbent amount provides fewer active sites for dye adsorption. Increasing the dose to 0.05 g L^−1^ significantly improves removal to 89.35%, as more adsorption sites become available. Beyond this point, removal efficiency exceeds 90% at 0.1 g L^−1^ and remains nearly constant up to 0.2 g L^−1^. This plateau indicates that 0.1 g L^−1^ is the optimal dosage; further increase yields negligible improvements, likely due to very low sorbate concentration offering a minimum concentration gradient for diffusion.

#### 3.5.3 Effect of adsorbate concentration

Fig. 4e illustrates the influence of initial RhB dye concentration on both removal efficiency and adsorption capacity (*q*_e_). As the dye concentration increases from 5 to 90 mg L^−1^, removal efficiency decreases, while adsorption capacity increases. At lower concentrations (<20 mg L^−1^), removal efficiency remains high (≥90%), indicating that the number of available adsorption sites are sufficient to capture most dye molecules present. However, at higher concentrations, removal efficiency declines to 72.44% at 30 mg L^−1^, 59.14% at 50 mg L^−1^, and 42.62% at 90 mg L^−1^. This reduction is due to the progressive saturation of available adsorption sites, which limits the fraction of dye removed from solution. In contrast, adsorption capacity (*q*_e_), defined as the mass of dye adsorbed per unit mass of adsorbent, increases substantially with concentration—from 44.19 mg g^−1^ at 5 mg L^−1^ to 383.55 mg g^−1^ at 90 mg L^−1^. This trend arises because higher initial concentrations provide a greater driving force for mass transfer, allowing each gram of adsorbent to hold more dye even though the overall percentage removal decreases. Thus, low initial concentrations favor high removal efficiency, whereas high initial concentrations maximize adsorption capacity, reflecting different aspects of adsorption performance.

#### 3.5.4 Adsorption kinetics

To comprehend the adsorption behavior of RhB onto the MOSP, which is the best sample in terms of adsorption, the experimental data is examined using several kinetic models. eqn (3)–(6) are used to calculate the various kinetic parameters, which are summarized in Table 2. The regression coefficient (*R*^2^) serves as a standard to evaluate the fitness of each model. The pseudo first order (PFO) model (Fig. 4f) exhibits poor agreement, with an *R*^2^ of 0.7568 and a considerable divergence between the calculated and experimental *q*_e_ values (31.60 and 99 mg g^−1^, respectively). This demonstrates that adsorption does not arise from physisorption kinetics, demonstrating that the PFO model does not accurately elucidate the rate-determining step. Conversely, the pseudo-second order (PSO) model (Fig. 4g) provides the best fit for the MOSP, with the highest correlation coefficient value of 0.9977 and close agreement between calculated and experimental *q*_e_ values (100 and 99 mg g^−1^, respectively). This indicates that adsorption is predominantly controlled by chemisorption. The non-linear fitting of the PFO, PSO, IPD, and Elovich model along with the experimental data is shown in Fig. 4j. Among all the models applied, the kinetic analysis strongly suggests that adsorption of RhB on MOSP is predominantly driven by chemisorption mechanisms, facilitated by electrostatic attraction. These observations are in close agreement with the findings of Inyinbor *et al.*,^77^ where the dominant mechanism is chemical adsorption followed by surface heterogeneity, confirming that SO_3_H and MoS_2_ work synergistically to provide rapid uptake and high affinity of RhB molecules.

**Table 2:** Comparative analysis of PFO, PSO, IPD, and Elovich kinetic models

Types of model	Parameter	Value
PFO	Experimental *q*_e_ (mg g^−1^)	99.00
	Calculated *q*_e_ (mg g^−1^)	31.60
	Rate constants (min^−1^)	1.75 × 10^−5^
	*R* ^2^	0.7568
PSO	Experimental *q*_e_ (mg g^−1^)	99.00
	Calculated *q*_e_ (mg g^−1^)	100
	Rate constants (g mg^−1^ min^−1^)	0.0026
	*R* ^2^	0.9977
IPD	*K*_id_ (mg g^−1^ min^−0.5^)	5.2909
	C	41.58
	*R* ^2^	0.6558
Elovich	α (mg g^−1^ min^−1^)	58.927
	*β* (g mg^−1^)	0.0609
	*R* ^2^	0.8922

#### 3.5.5 Adsorption isotherm

**Fig. 5:**
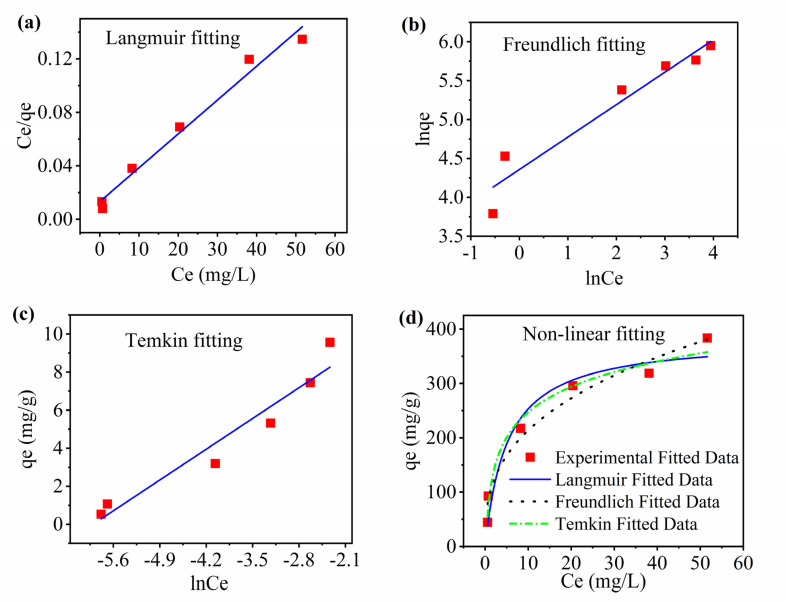
Adsorption isotherms (a) Langmuir linear fitted model, (b) Freundlich linear fitted model, (c) Temkin linear fitted model, and (d) non-linear curve fitted Langmuir, Freundlich, and Temkin model (Co = 10 mg L^−1^, contact time = 60 min, shaking speed = 250 rpm, S : L ratio = 0.1 g L^−1^).

The adsorption isotherms are used to explain the equilibrium relation between the amount of adsorbate adsorbed per gram of adsorbent and the concentration of adsorbate in solution.^78,79^ To elucidate the interaction between RhB and MOSP, the equilibrium data is analyzed using Langmuir, Freundlich, and Temkin isotherm models. The corresponding parameters and Langmuir separation factor (*R*_L_) are calculated, which are presented in Table 3, to assess the nature of the adsorption process and the applicability of each model in explaining the equilibrium data. Among the non-linear curve fitted (Fig. 5d) models, and the linear fitted, Langmuir isotherm (Fig. 5a) exhibited the highest coefficient of determination (*R*^2^ = 0.9814), indicating that RhB adsorption predominantly follows a monolayer adsorption mechanism, where each molecule occupies a distinct site without interaction with neighboring adsorbates. This behavior is consistent with chemisorption. The higher *Q*_max_ value derived from the Langmuir model fitting further demonstrates the high dye uptake capacity of the MOSP, which can be ascribed to the synergistic contribution of both the SO_3_H groups and MoS_2_ nanostructure. The *R*_L_ value (0.622) calculated from eqn (8) validates the assumption that the adsorption process is advantageous over the concentration range studied.^77^ The Freundlich adsorption intensity constant (N) further suggests favourable adsorption. Additionally, the Temkin binding constant (*A*_T_ = 368.30 L^−1^ g) confirms a strong interaction attributable to electrostatic attraction and π–π stacking between the conjugated structure of MoS_2_ and aromatic rings of RhB. The adsorption isotherm analysis reveals a complex adsorption mechanism. The best fit of the Langmuir model explains that the process is mainly monolayer in nature, indicating chemisorption.

**Table 3:** Parameters obtained from Langmuir, Freundlich, and Temkin isotherm for the adsorption of RhB onto MOSP

Type of model	Parameter	Value
Langmuir	*Q*_max_ (mg g^−1^)	400
	*K*_L_ (L mg^−1^)	0.1893
	*R*_L_	0.6221
	*R* ^2^	0.9814
Freundlich	*K*_F_ (mg g^−1^)	78.100
	N	2.3980
	*R* ^2^	0.9317
Temkin	*A*_T_ (L g^−1^)	368.30
	*B*_T_ (J mol^−1^)	2.3099
	*R* ^2^	0.9375

#### 3.5.6 Thermodynamic studies

**Fig. 6:**
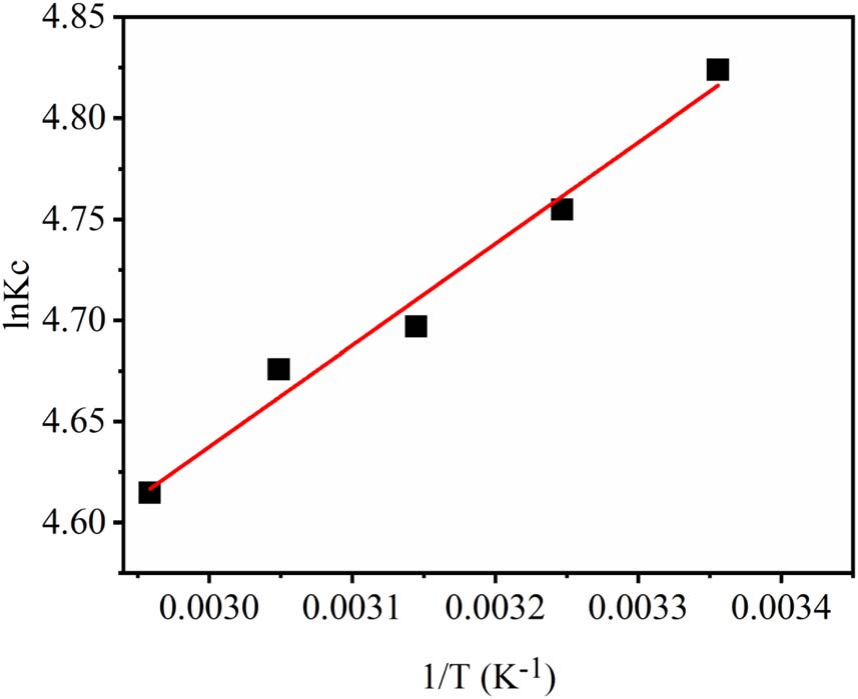
Van't Hoff plot for RhB adsorption onto MOSP (Co = 10 mg L^−1^, contact time = 60 min, shaking speed = 250 rpm, S : L ratio = 0.1 g L^−1^).

Thermodynamic parameters, such as standard enthalpy change (Δ*H*°) and standard entropy change (Δ*S*°), are calculated using van't Hoff equation (eqn (11)). The standard Gibbs free energy change (Δ*G*°) is computed using the standard Gibbs free energy equation (eqn (12)). The results are summarized in Table 4 and the corresponding van't Hoff plot is shown in Fig. 6. The value of Δ*H*° (−4.17 kJ mol^−1^) is negative, indicating the exothermic nature of the adsorption process, as the interaction between the adsorbent and adsorbate is energetically favourable, and the system releases heat during their interaction. The magnitude of Δ*H*° advocates that the process may involve electrostatic attraction between the SO_3_H groups and the RhB molecules, likely augmented by surface complexation with MoS_2_ nanostructures. The positive value of the Δ*S*° (0.02 kJ mol^−1^ k^−1^) implies that during the adsorption process, there is an increase in disorder at the solid–liquid interface. Additionally, the negative values of Δ*G*° at all temperatures show the spontaneity of the adsorption process. The calculated values of Δ*H*°, Δ*S*°, and Δ*G*° substantiate that adsorption of RhB by MOSP is a strongly exothermic process, accompanied by an increase in randomness and a spontaneous process. The higher adsorption efficiency of MOSP is observed at a lower temperature (298 K).

**Table 4:** Thermodynamic parameters of the adsorption of RhB onto MOSP

Δ*H*° (kJ mol^−1^)	Δ*S*° (kJ mol^−1^ K^−1^)	Temperature (K)	Δ*G*° (kJ mo1^−1^)
−4.1765	0.02	298	−11.93
		308	−12.06
		318	−12.45
		328	−12.71
		338	−12.97

#### 3.5.7 Effect of multiple metal ions on dye adsorption

**Fig. 7:**
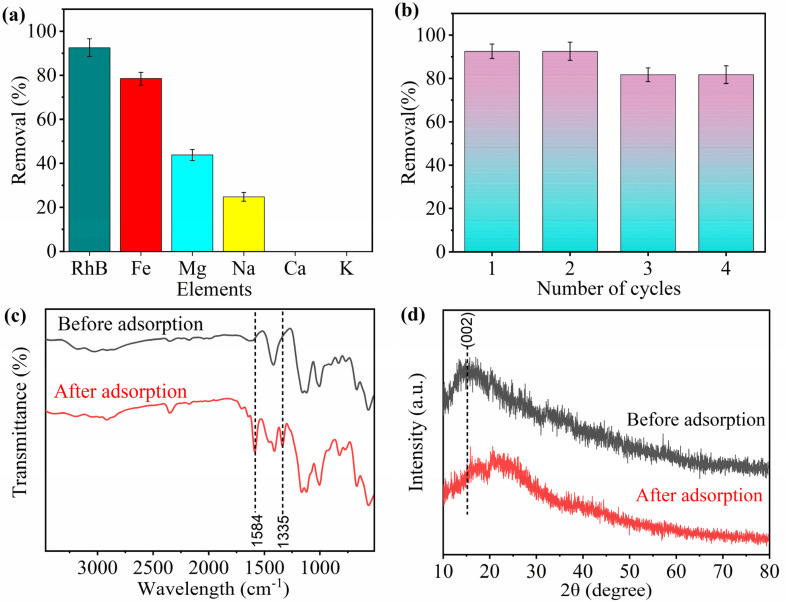
Effect of metal ions on RhB adsorption by MOSP (a), reusability of MOSP for RhB removal (b), MOSP (Co = 10 mg L^−1^, contact time = 60 min, shaking speed = 250 rpm, S : L ratio = 0.1 g L^−1^), and post-adsorption characterization: FTIR spectra of MOSP and RhB-MOSP (c), and XRD patterns of MOSP and RhB-MOSP (d).

Under real-world conditions, several interfering ions might exist in the system. Therefore, to examine the effect of multiple metal ions on RhB removal, an equimolar mixture is designed containing monovalent ions (Na and K), divalent ions (Fe, Mg, and Ca), and RhB molecules, and results are shown in Fig. 7a. The RhB shows highest removal efficiency ≥90%, sustaining the strong affinity of MOSP for RhB. This indicates that the interaction between the RhB molecules and the MOSP's surface is highly favourable. Moreover, the surface area of MoS_2_ improves this interaction and facilitates adsorption. Fe^2+^ and Mg^2+^ revealed removal around 80% and 45% due to their higher charge density compared to Ca^2+^, K^+^, and Na^+^. The highest removal of RhB despite the presence of monovalent and divalent interfering ions indicates that the MOSP exhibits high selectivity for RhB molecules.

#### 3.5.8 Regeneration and reusability

The reusability of an adsorbent is important to consider for mitigating secondary waste generation and reducing the process cost. In the removal of organic dyes, MoS_2_ exhibits excellent reusability.^80^ Under optimum conditions, MOSP is used for RhB removal; subsequently, the used material is collected and regenerated by adding it to a 0.1 M HCL solution and stirring for 35 min. Afterward, MOSP is thoroughly washed multiple times with DI water. The adsorption–desorption sequence of MOSP for RhB is examined over four cycles, and results are presented in Fig. 7b. After each cycle, the adsorbent material is separated by centrifugation and filtration, followed by washing with 0.1 M HCl, later with deionized water, and adjusted pH to 3. After that material is dried at 60 °C, before used for the next cycle. The removal efficiency reaches ≥90% in the first two cycles, indicating that the adsorbent retained its adsorption capacity after initial use. A slight decline in adsorption efficiency is observed in the third and fourth cycles; however, the removal performance still remains ≥80%, exhibiting the adsorbent's good regeneration potential and stability. This slight decline may be attributed to the surface fouling, incomplete desorption leading to the saturation of active sites, or minor leaching of MoS_2_ during subsequent washing steps.

#### 3.5.9 Comparison with other adsorbents

The adsorption capacity of MOSP is compared against a range of other reported adsorbents (Table 5), including metal oxides, activated carbons, clays, polymer composites, MOF-based hybrids, MoS_2_ nanopowder, and different MoS_2_-based composites. MOSP exhibits an exceptional adsorption capacity of 400 mg g^−1^, outperforming many other reported adsorbents.

**Table 5:** Comparison of various adsorption capacities of different adsorbents for the adsorption of RhB in terms of their adsorption capacities

Adsorbents	Co (mg L^−1^)	Adsorption capacity (mg g^−1^)	References
Palm shell-based activated carbon	20–100	29.98	81
C-carnauba-CaCl_2_	65–140	39.22	82
Sodium montmorillonite	100–5000	42.19	83
Kaolinite	20–100	46.10	84
MoS_2_-PDA@FRC (MoS_2_ modified biochar)	0–140	58.11	26
MRM-PS (modified red mud and polystyrene)	10–150	59.37	85
Graphene oxide/beta zeolite composite materials	20–80	64.47	86
Fe_3_O_4_/poly(St-*co*-MAA) particles	50–300	69.54	87
Zn/Co carbon composite	10–200	101.93	88
*Aleurites moluccana* (WAM)	50–300	117.00	89
MoS_2_	1–40	136.99	25
Zinc chloride-activated carbon	5–400	175.00	90
Fe_3_O_4_@N-Mc composite	16–50	178.80	91
MoS2 nanopowder	100–1300	210.24	27
MIL-68(In)NH_2_/graphite-oxide composites	2–200	267.00	92
Polar-modified-post-cross-linked resins PDBpc	208–1042	328.70	93
MoS_2_/MIL-101 hybrid	5–300	344.80	21
Ni/PC-CNT composites	20–350	395.00	94
MOSP	5–90	400.00	This study

This superior performance can be ascribed to the synergistic effect between the MoS_2_ and sulfonated polystyrene. The presence of the SO_3_H group on polystyrene resin facilitates electrostatic attraction towards the cationic dye (RhB) molecules, and MoS_2_ offers a maximum number of active sites, exhibiting a higher surface area for π–π interactions and hydrogen bonding. This dual mechanism not only increases the number of accessible binding sites but also facilitates dye uptake.

In addition to its remarkable adsorption performance, MOSP is economically feasible, using readily available, inexpensive reagents and a simple hydrothermal process with a regular laboratory setup. No specialized equipment or costly catalysts are needed, and the synthesis is facile and reproducible. The material can be regenerated and reused many times with minimal loss in effectiveness, further strengthening its cost-effectiveness. The elevated adsorption capacity and cost effectiveness observed for MOSP underscore its feasibility for real-world application in industrial wastewater processing. Its ability to surpass a broad range of existing materials suggests that it holds substantial potential for expandable implementation in dye-laden effluent remediation, especially in textiles and chemical manufacturing sectors seeking efficient, economical, and environmentally viable treatment solutions.

#### 3.5.10 Possible adsorption mechanism

**Fig. 8:**
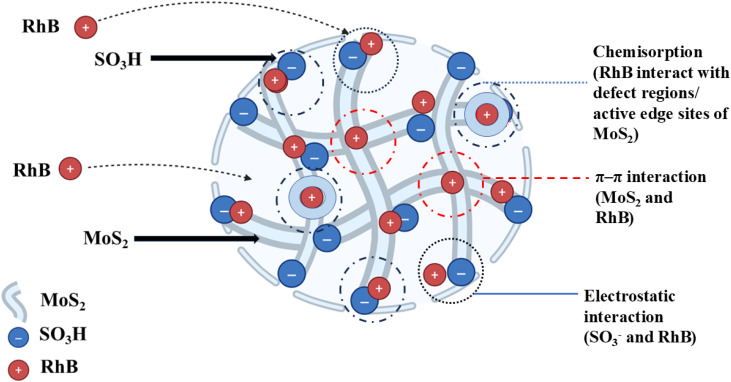
Schematic illustration of the adsorption mechanism.

The adsorption of RhB onto MOSP likely to have followed a synergistic multi-interaction mechanism (Fig. 8). Primarily, electrostatic attraction plays a crucial role, attributable to the negatively charged (SO_3_^−^) groups resulting from sulfonation. These groups exhibit a strong affinity for cationic molecules (RhB), facilitating fast initial uptake. Concurrently, the conjugated π-system of MoS_2_ promotes π–π stacking interaction with the RhB's aromatic ring, enhancing selectivity and binding affinity. As adsorption continues, RhB molecules strongly interact with defect regions and active edge sites on the MoS_2_ surface, resulting in chemical adsorption through the donor–acceptor mechanism. This supports the energy-driven adsorption behaviour presented by thermodynamic data. The MOSP structure creates a heterogeneous surface with different energy binding sites. Although the equilibrium is achieved within a monolayer. However, the multi-step mechanism governs the pathway of the adsorption process that follows an electrostatic and π–π interaction, facilitated by chemisorption.^21,38^

#### 3.5.11 Post adsorption characterization

The structural and vibrational characterization of MOSP before and after adsorption of RhB indicates the pronounced changes and material's stability in the composite system. In the FTIR spectra (Fig. 7c) after the adsorption of RhB, the peak intensity at 1575 cm^−1^ increases, and a new peak appears at approximately 1335 cm^−1^, supported by the FTIR spectrum of RhB (Fig. S9). This change is due to additional fused aromatic rings present in RhB coupled with the phenyl groups (π– π stacking), as RhB contains a xanthene scaffold fused with two benzene rings, thereby rendering it strongly conjugated, and such systems can resonate with the aromatic rings in the host matrix. This confirms the existence of RhB on the MOSP surface.^77^ Importantly, no significant changes are seen in SO_3_^−^-stretches, demonstrating that SO_3_H groups are involved in electrostatic interactions with RhB. However, it is not chemically altered, which verifies a stable adsorption mechanism. In the XRD patterns (Fig. 7d) after the adsorption of RhB, the (002) reflection moves towards a marginally higher angle, which aligns with a decrease in interlayer spacing. Overall, the diffraction intensity of all peaks decreased, suggesting partial surface coverage due to adsorption. The evolution of FTIR and XRD features provides compelling evidence of RhB adsorption onto the MoS_2_ surface, rather than solely residing in the sulfonated polystyrene matrix.

## 4. Conclusion

This study explores the successful synthesis of MoS_2_-integrated sulfonated polystyrene divinylbenzene composite (MOSP) as a highly efficient adsorbent for the removal of RhB dye from an aqueous solution. A comparative evaluation with MoS_2_-integrated pristine polystyrene composite (MOP) substantiates the exceptional performance of MOSP, associated with the maximum loading of MoS_2_ by SO_3_H groups. The prepared adsorbent materials are investigated by SEM, XRD, Raman, FTIR, CHNS elemental analysis, BET, and TGA techniques. A UV-visible spectrophotometer and an ICP-OES analyzer are also employed to detect the amount of RhB and interfering metal ions in the RhB solution. Batch adsorption experiments are used to evaluate the effect of pH, contact time, dye concentration, adsorbent dosage, temperature, and the interference of multiple ions. pH 3, and 0.1 g L^−1^ were identified as an optimum pH and adsorbent dose for efficient adsorption of RhB. The kinetic analysis reveals chemisorption as the dominant mechanism, consistent with the pseudo-second order model, and the material exhibits rapid kinetics, as 79% of the dye was removed after 15 min, and equilibrium was achieved within one hour. MOSP exhibits a higher adsorption capacity of 400 mg g^−1^ for RhB based on the Langmuir adsorption model under optimum conditions. The cumulative effects of MoS_2_ and SO_3_H groups have facilitated the higher adsorption capacity, making MOSP a viable candidate for removing RhB from industrial effluents. Thermodynamic studies reveal that the adsorption process is spontaneous and exothermic. In the presence of alkali, alkaline, and transition metal ions, MOSP maintains the removal efficiency of >90% for RhB. MOSP, prepared by a simple synthesis procedure, exhibits high removal efficiency, rapid kinetics, high adsorption capacity, cost-effectiveness, and high stability. Overall, the MOSP composite offers a scalable and promising solution for the mitigation of textile dye-polluted water. However, the material may be effective only for cationic dyes. Further studies are needed to analyse the performance of the adsorbent in real wastewater and to optimize its large-scale synthesis.

## Conflicts of interest

There are no conflicts to declare.

## Supplementary Material

RA-016-D5RA07598J-s001

## Data Availability

Data will be available on request. Supplementary information (SI) is available. See DOI: https://doi.org/10.1039/d5ra07598j.

## References

[cit1] Robinson T., McMullan G., Marchant R., Nigam P. (2001). Bioresour. Technol..

[cit2] Rasalingam S., Peng R., Koodali R. T. (2015). Appl. Catal., B.

[cit3] Richardson S. D., Willson C. S., Rusch K. A. (2004). Groundwater.

[cit4] Xiang P., Tang C., Ma K., Li X. (2025). J. Water Proc. Eng..

[cit5] Rochat J., Demenge P., Rerat J. (1978). Eur. J. Toxicol..

[cit6] Jain R., Mathur M., Sikarwar S., Mittal A. (2007). J. Environ. Manage..

[cit7] Jiang X., Tang C., Li X., Chen Z. (2025). Chem. Eng. J..

[cit8] Xiang P., Tang C., Ma K., Li X. (2025). Desalination.

[cit9] Qing W., Xiang P., Tang C., Li X., Chen Z. (2025). Chem. Eng. J..

[cit10] Al-Gheethi A. A., Azhar Q. M., Kumar P. S., Yusuf A. A., Al-Buriahi A. K., Mohamed R. M. S. R., Al-Shaibani M. M. (2022). Chemosphere.

[cit11] Gupta V. K. (2009). J. Environ. Manage..

[cit12] Demirbas A. (2009). J. Hazard. Mater..

[cit13] Rafatullah M., Sulaiman O., Hashim R., Ahmad A. (2010). J. Hazard. Mater..

[cit14] Vakili M., Rafatullah M., Salamatinia B., Abdullah A. Z., Ibrahim M. H., Tan K. B., Gholami Z., Amouzgar P. (2014). Carbohydr. Polym..

[cit15] Yagub M. T., Sen T. K., Afroze S., Ang H. M. (2014). Adv. Colloid Interface Sci..

[cit16] Xia T., Lin Y., Li W., Ju M. (2021). Chin. Chem. Lett..

[cit17] Wang X., Tuo Y., Zhou Y., Wang D., Wang S., Zhang J. (2021). Chem. Eng. J..

[cit18] Haleem A., Shafiq A., Chen S.-Q., Nazar M. (2023). Molecules.

[cit19] Amaral L. O., Daniel-da-Silva A. L. (2022). Molecules.

[cit20] Alarifi I. M., Al-Ghamdi Y. O., Darwesh R., Ansari M. O., Uddin M. K. (2021). J. Mater. Res. Technol..

[cit21] Yang C., Cheng J., Chen Y., Hu Y. (2017). J. Colloid Interface Sci..

[cit22] Pirarath R., Shivashanmugam P., Syed A., Elgorban A. M., Anandan S., Ashokkumar M. (2021). Front. Environ. Sci. Eng..

[cit23] Zeng M., Yang B., Yan H., Qu H., Hu Y. (2020). Chem. Phys. Lett..

[cit24] Wang Z., Zhu W., Qiu Y., Yi X., von dem Bussche A., Kane A., Gao H., Koski K., Hurt R. (2016). Chem. Soc. Rev..

[cit25] Li Z., Meng X., Zhang Z. (2019). Mater. Res. Bull..

[cit26] Ma R., Nie D., Sang M., Wang W., Nie G. (2023). Bioresour. Technol..

[cit27] Fang Y., Qu Q. (2023). J. Phys.:Conf. Ser..

[cit28] Zhou W., Yin Z., Du Y., Huang X., Zeng Z., Fan Z., Liu H., Wang J., Zhang H. (2013). Small.

[cit29] Fu H., Yu K., Li H., Li J., Guo B., Tan Y., Song C., Zhu Z. (2015). Dalton Trans..

[cit30] Shao J.-H., Deng Y.-M., Song L., Batur A., Jia N., Liu D.-Y. (2016). LWT–Food Sci. Technol..

[cit31] Khaliq K., Anjum M. A. R., Shahida S., Akhtar R., Khan A., Shafiq M. A., Rafiq I., Rehan M., Qureshi R. N., Iqbal S. (2025). RSC Adv..

[cit32] Liu C., Zeng S., Yang B., Jia F., Song S. (2019). J. Mol. Liq..

[cit33] Parida K., Mishra K. G., Dash S. K. (2012). J. Hazard. Mater..

[cit34] Yadav V., Rajput A., Kulshrestha V. (2020). J. Membr. Sci..

[cit35] Yeddou N., Bensmaili A. (2005). Desalination.

[cit36] Wu F.-C., Tseng R.-L., Juang R.-S. (2009). Chem. Eng. J..

[cit37] Akhtar R., Latif S., Shah S. A. A., Saeed S., Aziz A. (2023). Nucl. Eng. Technol..

[cit38] Weng X. C., Ajmal M., Shehzad H., Chen J., Farooqi Z. H., Liu Z., Sharif A., Ahmed E., Zhou L., Xu L. (2024). Int. J. Biol. Macromol..

[cit39] Anjum M. A. R., Iqbal S., Toba Z., Javaid S., Jamal A., Shafique M. A., Ullah M. S. (2023). New J. Chem..

[cit40] Elabd A., Zidan W., Abo-Aly M., Bakier E., Attia M. (2014). J. Environ. Radioact..

[cit41] Yuan H., Kalfas G., Ray W. (1991). J. Macromol. Sci. Polym. Rev..

[cit42] Ali M. A., Mubarak M. F., Keshawy M., Zayed M. A., Ataalla M. (2022). Alexandria Eng. J..

[cit43] Yan S., Qiao W., He X., Guo X., Xi L., Zhong W., Du Y. (2015). Appl. Phys. Lett..

[cit44] Martin C., Ramirez L., Cuellar J. (2003). Surf. Coat. Technol..

[cit45] Chatti M., Gengenbach T., King R., Spiccia L., Simonov A. N. (2017). Chem. Mater..

[cit46] Rasamani K. D., Alimohammadi F., Sun Y. J. M. T. (2017). Mater. Today.

[cit47] Frey G. L., Tenne R., Matthews M. J., Dresselhaus M., Dresselhaus G. (1999). Phys. Rev. B:Condens. Matter Mater. Phys..

[cit48] Placidi M., Dimitrievska M., Izquierdo-Roca V., Fontané X., Castellanos-Gomez A., Pérez-Tomás A., Mestres N., Espindola-Rodriguez M., López-Marino S., Neuschitzer M. (2015). 2D Mater.

[cit49] Jia F., Wang Q., Wu J., Li Y., Song S. (2017). ACS Sustainable Chem. Eng..

[cit50] Robinson B. J., Giusca C. E., Gonzalez Y. T., Kay N. D., Kazakova O., Kolosov O. V. (2015). 2D Mater.

[cit51] Zhang S., Liu J., Ruiz K. H., Tu R., Yang M., Li Q., Shi J., Li H., Zhang L., Goto T. (2018). Mater. Today.

[cit52] Velea A., Buruiana A.-T., Mihai C., Matei E., Tite T., Sava F. (2024). Crystals.

[cit53] Tuschel D. (2015). J. Spectrosc.

[cit54] Lee C., Yan H., Brus L. E., Heinz T. F., Hone J., Ryu S. (2010). ACS Nano.

[cit55] Pimenta M. A., Del Corro E., Carvalho B. R., Fantini C., Malard L. M. (2015). Acc. Chem. Res..

[cit56] Mao Y., Dong N., Wang L., Chen X., Wang H., Wang Z., Kislyakov I. M., Wang J. (2020). J. Nanomater..

[cit57] Mahla S. K., Kesarwani S., Pal A. (2025). J. Alloys Compd..

[cit58] Sangeethavanathi S., Gowthaman P., Vigneswaran S., Sathishkumar M., Pal D. (2024). Mater. Sci. Res. India.

[cit59] Li H., Zhang Q., Yap C. C. R., Tay B. K., Edwin T. H. T., Olivier A., Baillargeat D. (2012). Adv. Funct. Mater..

[cit60] Ikram M., Tabassum R., Qumar U., Ali S., Ul-Hamid A., Haider A., Raza A., Imran M. (2020). RSC Adv..

[cit61] Singh V. K., Mukherjee B., Aravindh S. A., Das S. (2023). Appl. Surf. Sci..

[cit62] Zhao J., Zhang Z., Yang S., Zheng H., Li Y. (2013). J. Alloys Compd..

[cit63] Bhutto A., Vesely D., Gabrys B. (2003). J. Polym..

[cit64] Brijmohan S. B., Swier S., Weiss R., Shaw M. T. (2005). Ind. Eng. Chem. Res..

[cit65] Yang J. C., Jablonsky M. J., Mays J. W. (2002). Polym.

[cit66] Pandey S. K., Arunan E. (2021). ChemistrySelect.

[cit67] Hazarika M., Jana T., Compos J. (2013). Sci.

[cit68] Bao C., Guo Y., Song L., Hu Y. (2011). J. Mater. Chem..

[cit69] Zhao M., Ma X., Yan S., Xiao H., Li Y., Hu T., Zheng Z., Jia J., Wu H. (2020). Int. J. Hydrogen Energy.

[cit70] Hua M., Jiang Y., Wu B., Pan B., Zhao X., Zhang Q. (2013). ACS Appl. Mater. Interfaces.

[cit71] Zheng Q., Morgan R., Compos J. (1993). Mater.

[cit72] Suleiman D., Napadensky E., Sloan J. M., Crawford D. M. (2007). Thermochim. Acta.

[cit73] Jang B. N., Wilkie C. A. (2005). Polymer.

[cit74] Shakir K., Elkafrawy A. F., Ghoneimy H. F., Beheir S. G. E., Refaat M. (2010). Water Resour. Ind..

[cit75] Kisała J., Ferraria A. M., Mitina N., Cieniek B., Krzemiński P., Pogocki D., Nebesnyi R., Zaichenko O., Bobitski Y. (2022). RSC Adv..

[cit76] Wang X., Chen X., Peng Y., Pan J. (2019). RSC Adv..

[cit77] Inyinbor A., Adekola F., Olatunji G. A. (2016). Water Resour. Ind..

[cit78] Mumtaz K., Iqbal S., Shahida S., Shafique M. A., Wasim M., Ahmad B. (2021). Microporous Mesoporous Mater..

[cit79] Karthik R., Meenakshi S. (2014). JWPE.

[cit80] Mohammed R., Ali M. E. M., Abdel-Moniem S. M., Ibrahim H. S. (2022). Nano-Struct. Nano-Objects.

[cit81] Mohammadi M., Hassani A. J., Mohamed A. R., Najafpour G. D. (2010). J. Chem. Eng. Data.

[cit82] da Silva Lacerda V., López-Sotelo J. B., Correa-Guimarães A., Hernández-Navarro S., Sánchez-Báscones M., Navas-Gracia L. M., Martín-Ramos P., Martín-Gil J. (2015). J. Environ. Manage..

[cit83] Selvam P. P., Preethi S., Basakaralingam P., Thinakaran N., Sivasamy A., Sivanesan S. (2008). J. Hazard. Mater..

[cit84] Khan T. A., Dahiya S., Ali I. (2012). Appl. Clay Sci..

[cit85] Cao J., Wang Y., Yan Z., Li G. J. M. (2014). Nano Lett..

[cit86] Cheng Z.-L., Li Y.-X., Liu Z. (2017). J. Alloys Compd..

[cit87] Hayasi M., Karimi M. (2017). Polym. Bull..

[cit88] Zhao H., Wang Y., Zhao L. (2017). Eur. J. Inorg. Chem..

[cit89] Postai D. L., Demarchi C. A., Zanatta F., Melo D. C. C., Rodrigues C. A. (2016). Alexandria Eng. J..

[cit90] Zhi L. L., Zaini M. A. A. (2017). Water Sci. Technol..

[cit91] Liang T., Wang F., Liang L., Liu M., Sun J. (2016). J. Mater. Sci..

[cit92] Yang C., Wu S., Cheng J., Chen Y. (2016). J. Alloys Compd..

[cit93] Jiang X., Huang J. (2016). J. Colloid Interface Sci..

[cit94] Jin L., Zhao X., Qian X., Dong M. (2018). J. Colloid Interface Sci..

