# Supplementary material for: MoS2-functionalized sulfonated polystyrene for adsorption of rhodamine B
Source: RSC Adv. 2026 Jan 5;16(2):1051–67. doi: 10.1039/d5ra07598j (PMC12771072; doi:10.1039/d5ra07598j)
Supplement: RA-016-D5RA07598J-s001 [file RA-016-D5RA07598J-s001.pdf]

Supplementary Information (SI) for RSC Advances.
This journal is © The Royal Society of Chemistry 2026

Supplementary information

MoS_2-functionalized sulfonated polystyrene for adsorption of rhodamine B

Kashmala Khaliq,[a] Adil Khan,[b] Shabnam Shahida,[a*] Ramzan Akhtar,[b] Mohsin Ali Raza Anjum,[b] Iqra Rafiq,[c] Muhammad Rehan,[d] Rashid Nazir Qureshi,[e] Sajid Iqbal,[f*] Muhammad Saifullah[b*]

[a] Department of Chemistry, University of Poonch Rawalakot, AJK, Pakistan

[b] Chemistry Division, Pakistan Institute of Nuclear Science and Technology (PINSTECH), Nilore 45650, Islamabad, Pakistan

[c] Department of Chemistry, Government College University Faisalabad (GCUF), Faisalabad, Pakistan

[d] Photovoltaic Research Department, Korea Institute of Energy Research, Daejeon, South Korea

[e] Central Analytical Facility Division, Pakistan Institute of Nuclear Science and Technology (PINSTECH), Nilore 45650, Islamabad, Pakistan

[f] Department of Nuclear and Quantum Engineering, KAIST, 291 Deahak-ro, Yuseong-gu, Daejeon 34141, Republic of Korea

[*] Corresponding Authors:

S. Shahida (shabnamshahida01@gmail.com), S. Iqbal (sajid1@kaist.ac.kr), M. Saifullah (saifi.551@gmail.com)

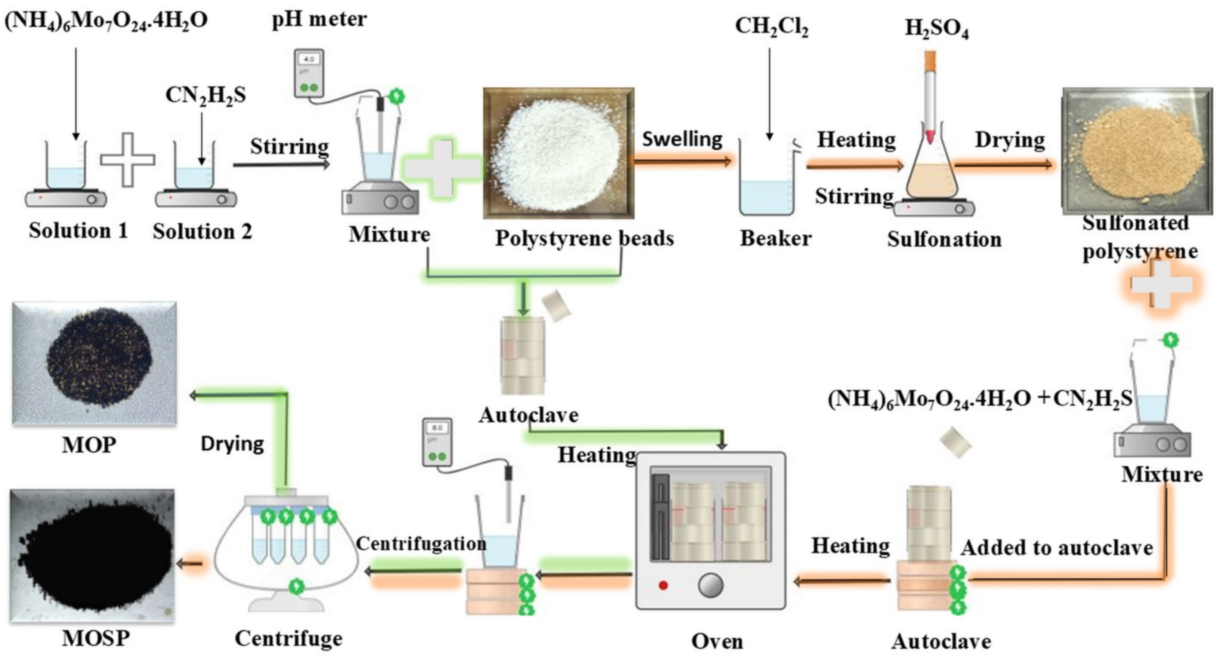

Fig. S1 Synthesis schematic illustration of MOP and MOSP.

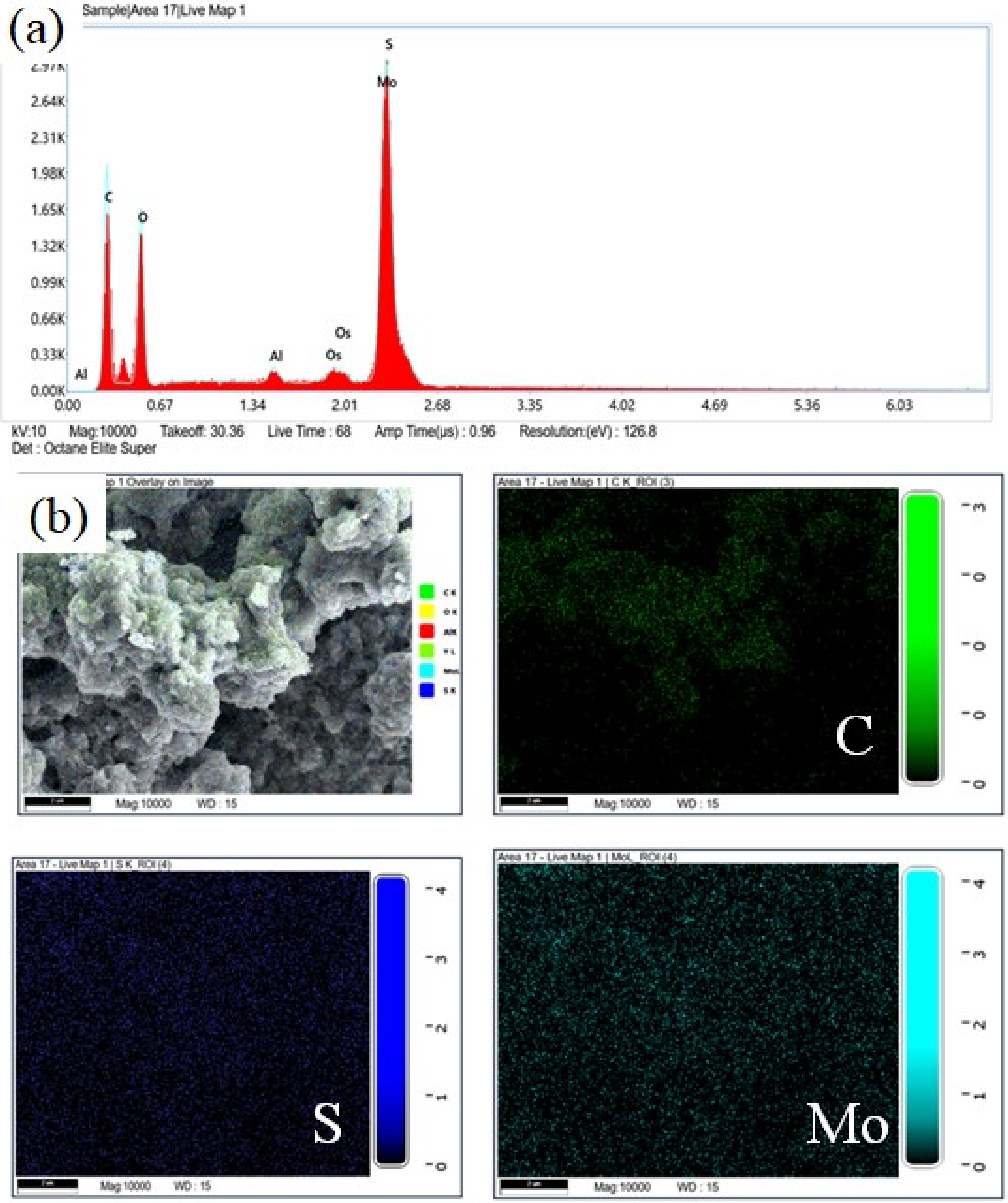

Fig. S2 (a) EDX spectrum and (b) elemental mapping analysis of MOSP.

Fig. S2. shows the EDX spectrum of the MOSP composite. The peaks of C, O, Mo, and S are clearly seen, although the overlapping of of Mo and S peaks occurs due to their close energy levels. The detected Al signal originates from the sample holder, whereas the presence of Os is ascribed to the sample coating used during adsorbent analysis. Furthermore, Fig. S1b presents elemental

mapping of MOSP, which validates the homogeneous and uniform distribution of Mo, S, and C throughout the MOSP matrix, demonstrating successful dispersion and incorporation of active components in the sulfonated polystyrene. Conversely, Fig. S2 shows elemental mapping of MOP, which confirms the non-uniform dispersion of Mo, S, and C throughout the MOP matrix.

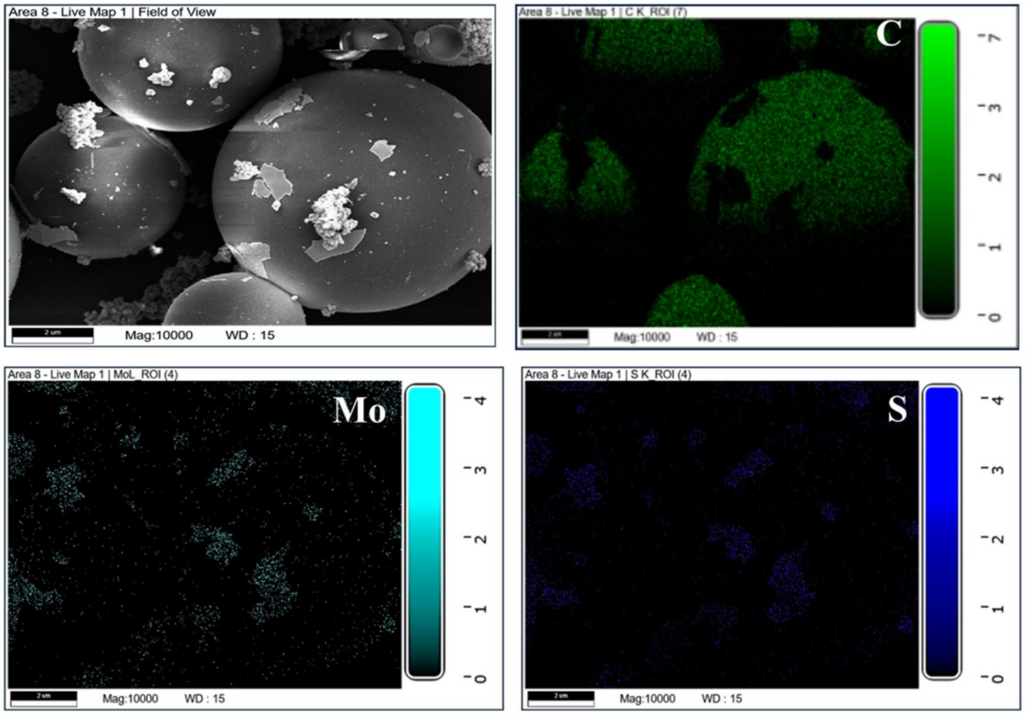

Fig. S3 Elemental mapping of MOP.

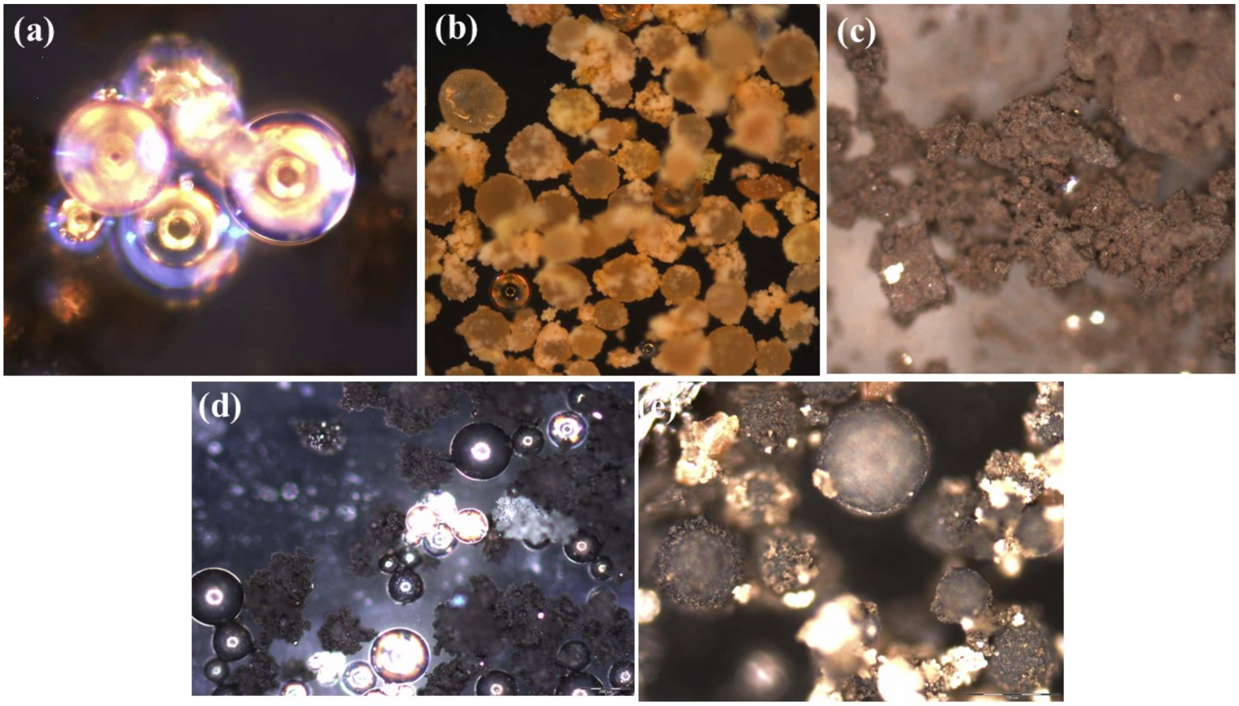

Fig. S4 Optical images of pristine polystyrene (a), sulfonated polystyrene (b), MoS_2 (c), MOP (d), and MOSP (e).

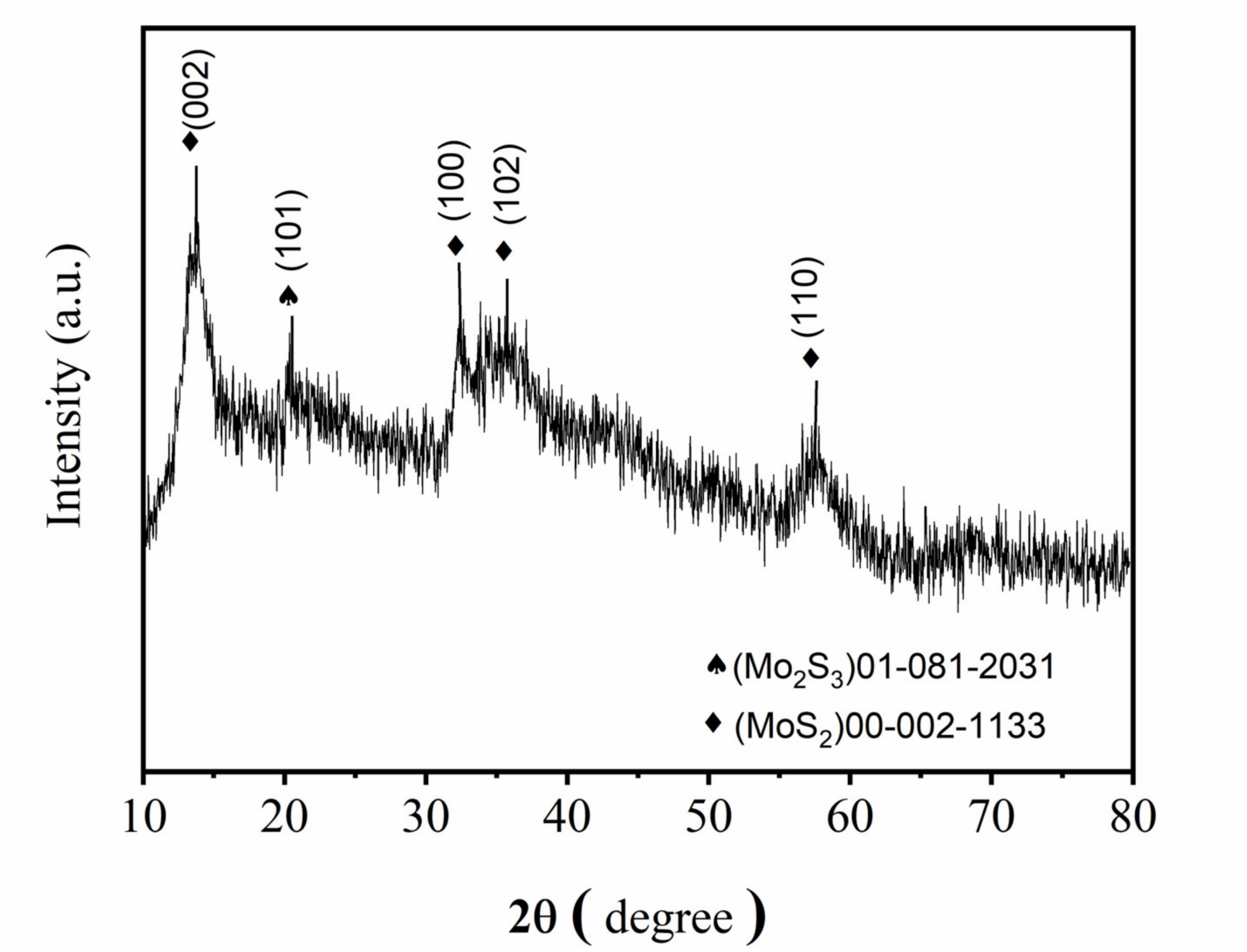

Fig. S5 XRD spectrum of pristine MoS_2.

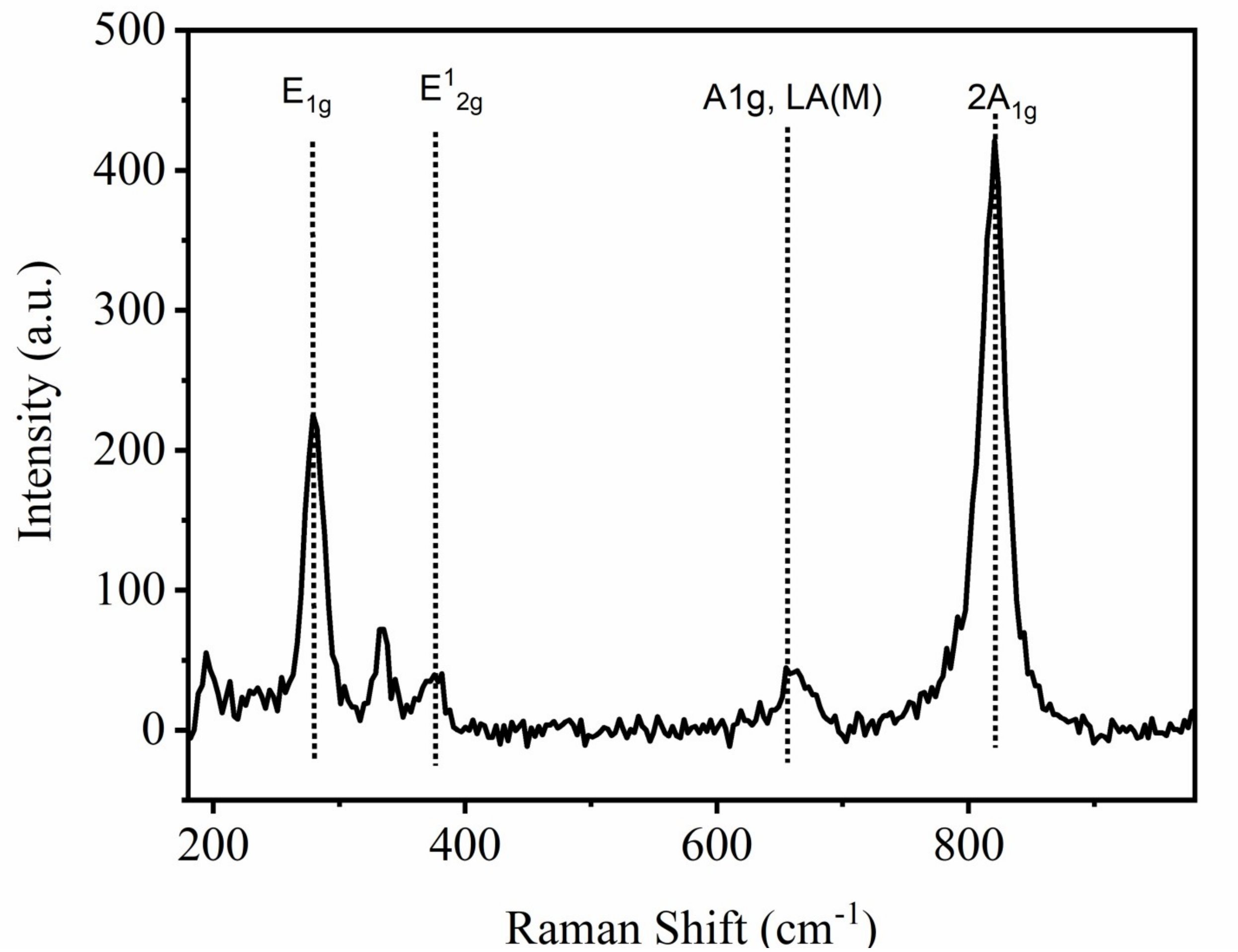

Fig. S6 Raman spectrum of pristine MoS_2.

Table S1 Physicochemical properties of MOP and MOSP

Adsorbent	MOP	MOSP
Incorporated material	MoS_2	
Host	Pristine polystyrene	Sulfonated polystyrene
BET surface area	1.6350 m^2/g	2.4068 m^2/g
Pore volume	0.0681 cm^3/g	0.0163 cm^3/g
Pore size	166.7 nm	27.0 nm

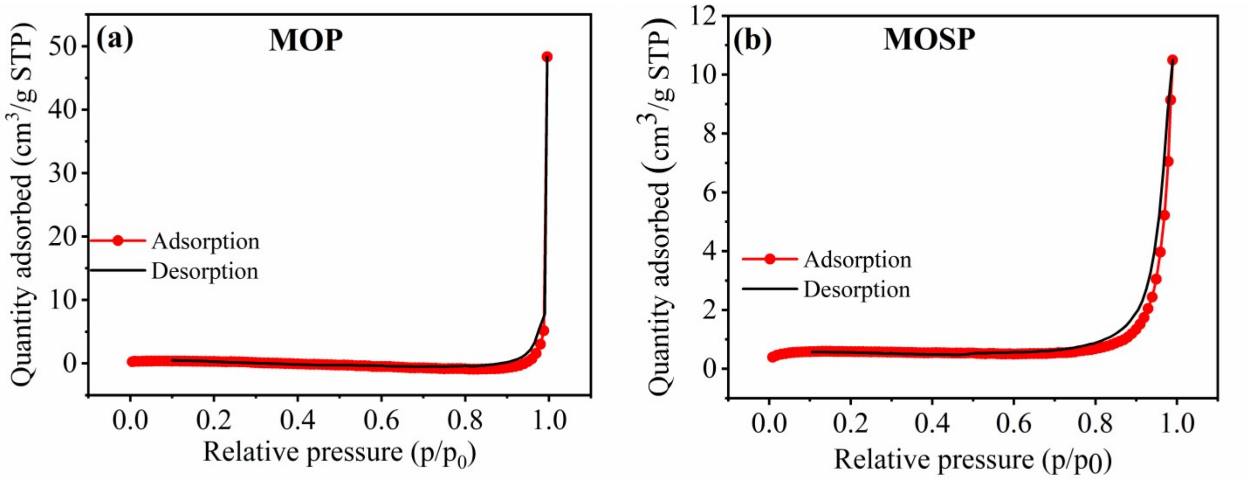

Fig. S7 N_2 adsorption and desorption of MOP (a) and MOSP (b).

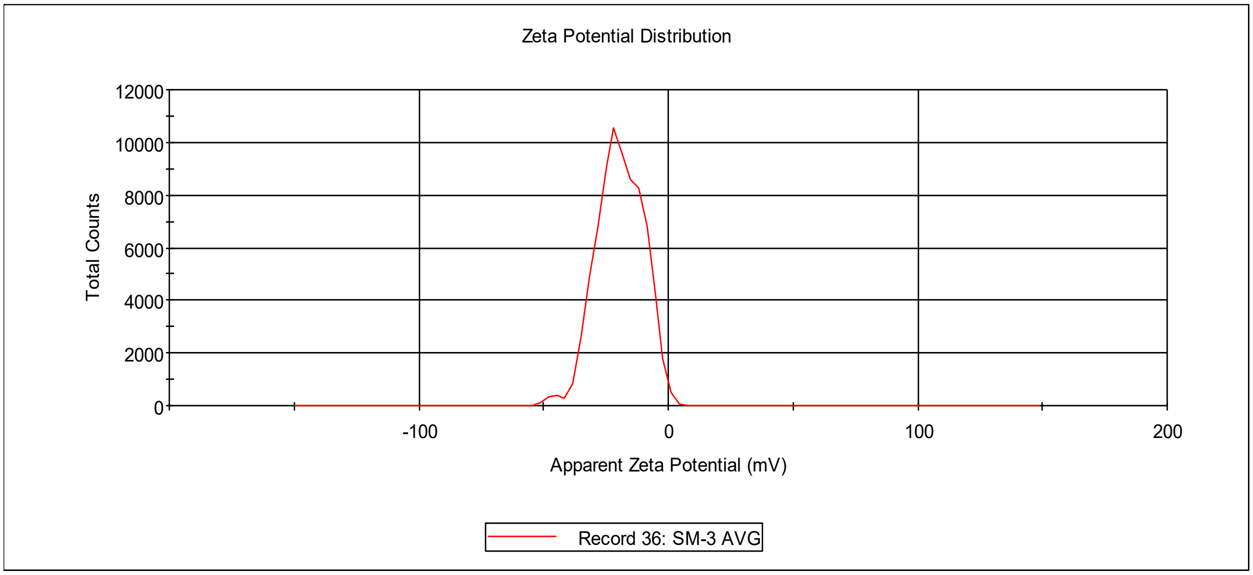

Fig. S8a Zeta potential of MOSP measured at pH 3.

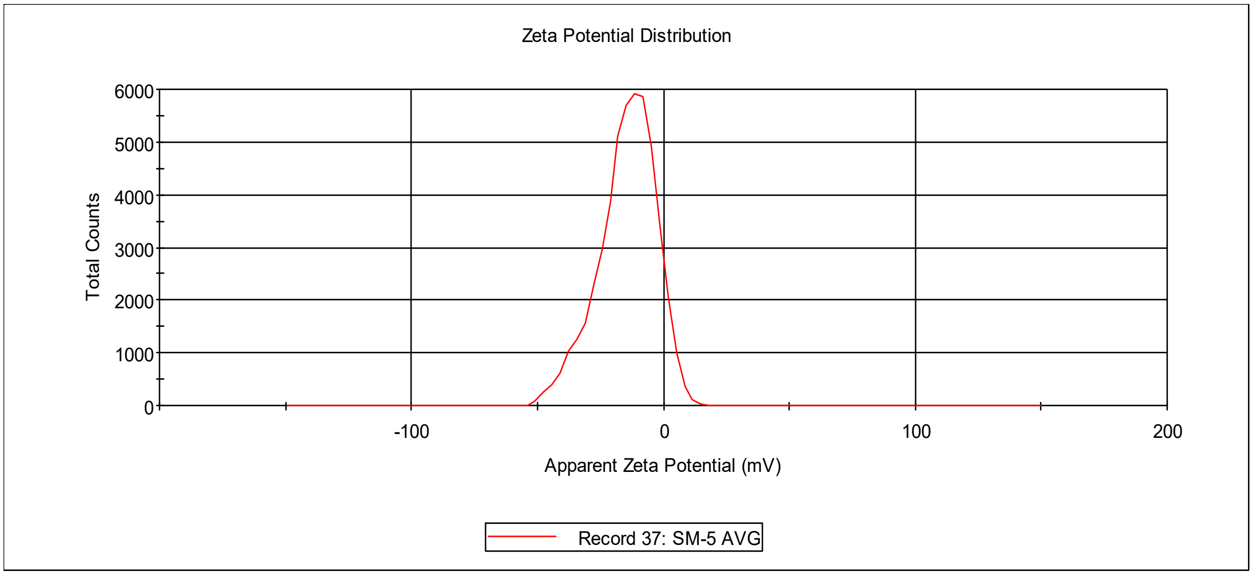

Fig. S8b Zeta potential of MOSP measured at pH 5.

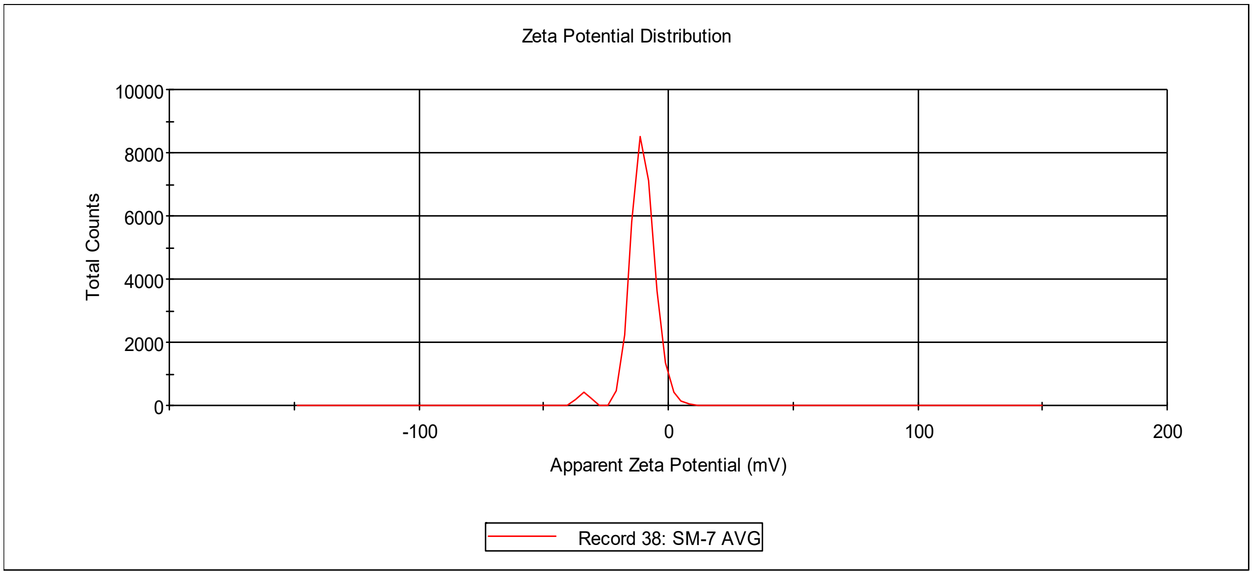

Fig. S8c Zeta potential of MOSP measured at pH 7.

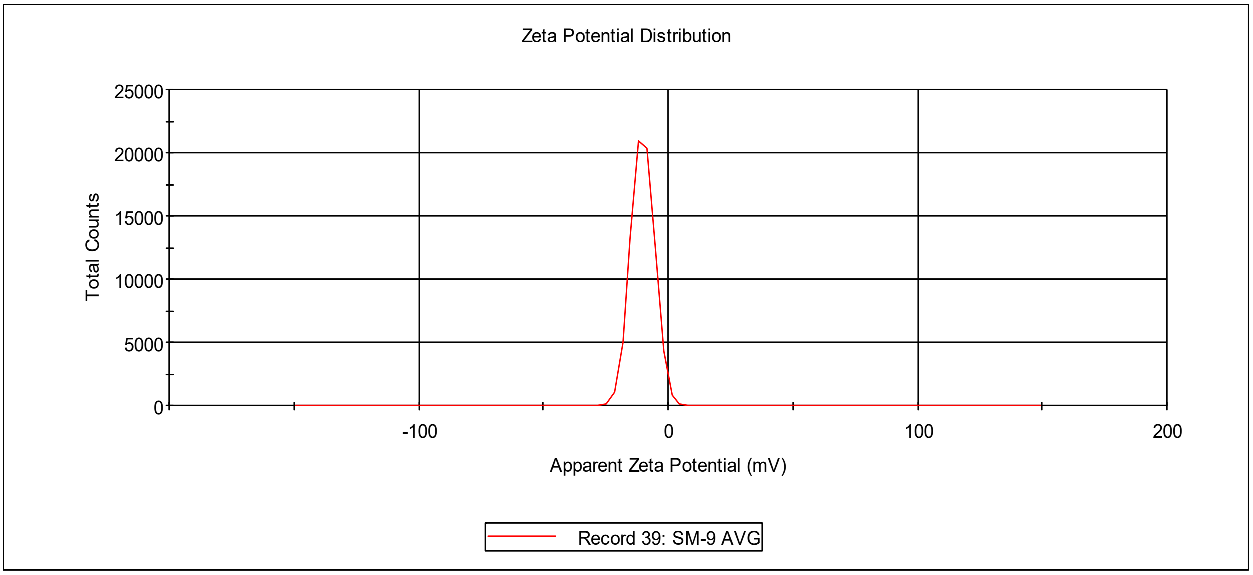

Fig. S8d Zeta potential of MOSP measured at pH 9.

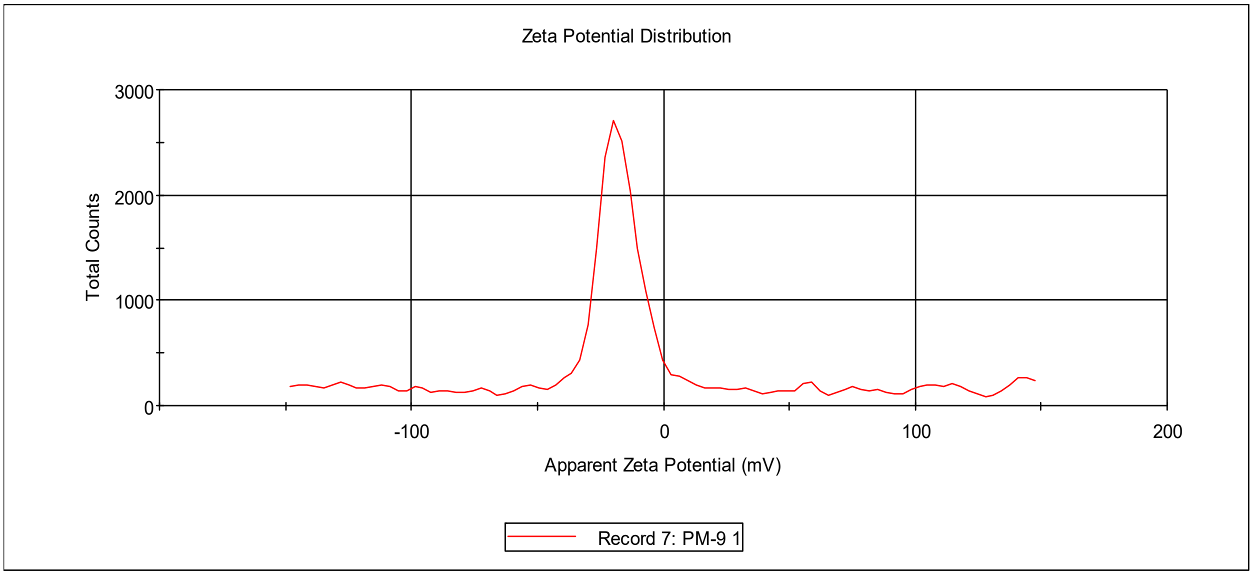

Fig. S8e Zeta potential of MOSP measured at pH 11.

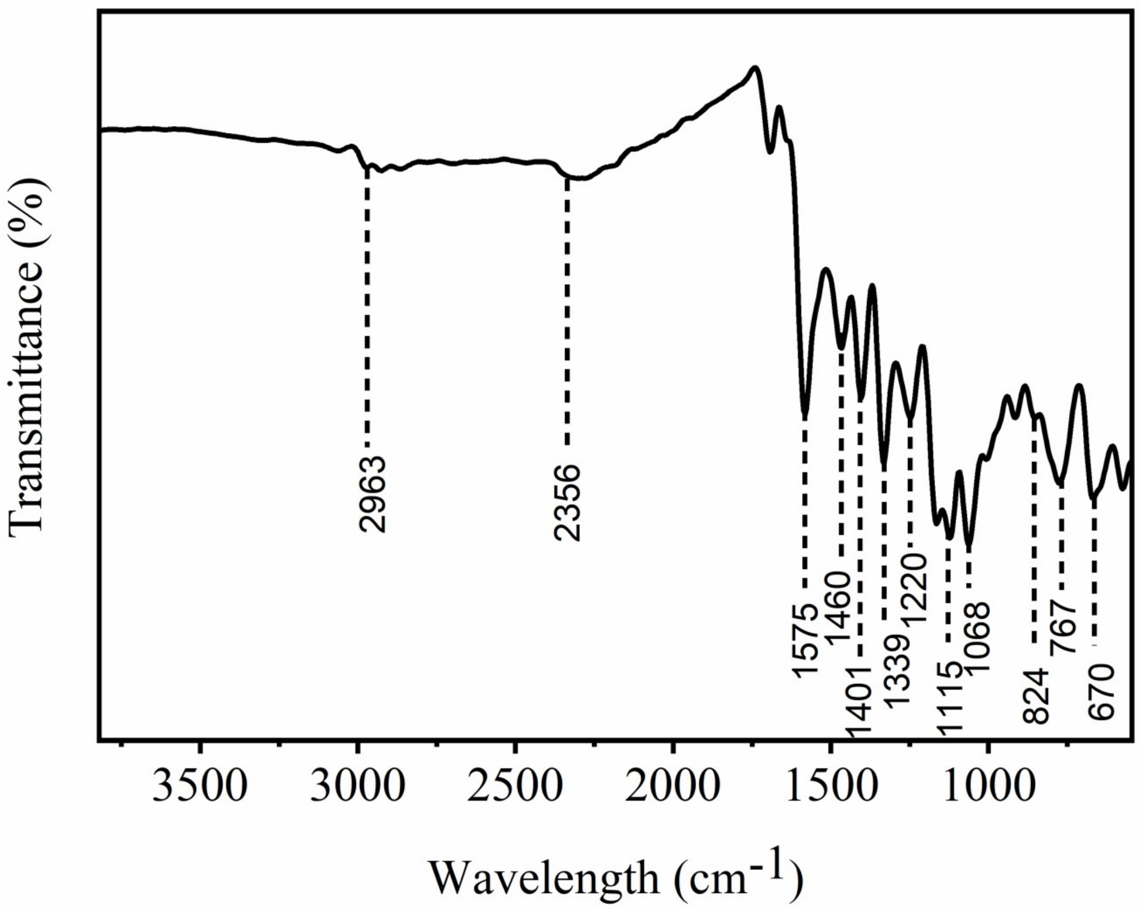

Fig. S9 FTIR spectrum of rhodamine B.
